# The Global Hepatitis B Virus Genotype Distribution Approximated from Available Genotyping Data

**DOI:** 10.3390/genes9100495

**Published:** 2018-10-15

**Authors:** Stoyan Velkov, Jördis J. Ott, Ulrike Protzer, Thomas Michler

**Affiliations:** 1 Institute of Virology, Technische Universität München/Helmholtz Zentrum München, Trogerstrasse 30, D-81675 München, Germany; stoyan.velkov@tum.de (S.V.); protzer@tum.de (U.P.); 2 Department of Epidemiology, Helmholtz Centre for Infection Research, D-38124 Braunschweig, Germany; Hannover Medical School, D-30625 Hannover, Germany; Joerdis.Ott@helmholtz-hzi.de; 3 German Center for Infection Research (DZIF), Munich Partner Site

**Keywords:** Hepatitis B virus, chronic hepatitis B, genotype, sequencing, molecular epidemiology

## Abstract

Hepatitis B virus (HBV) is divided into nine genotypes, A to I. Currently, it remains unclear how the individual genotypes contribute to the estimated 250 million chronic HBV infections. We performed a literature search on HBV genotyping data throughout the world. Over 900 publications were assessed and data were extracted from 213 records covering 125 countries. Using previously published HBV prevalence, and population data, we approximated the number of infections with each HBV genotype per country and the genotype distribution among global chronic HBV infections. We estimated that 96% of chronic HBV infections worldwide are caused by five of the nine genotypes: genotype C is most common (26%), followed by genotype D (22%), E (18%), A (17%) and B (14%). Genotypes F to I together cause less than 2% of global chronic HBV infections. Our work provides an up-to-date analysis of global HBV genotyping data and an initial approach to estimate how genotypes contribute to the global burden of chronic HBV infection. Results highlight the need to provide HBV cell culture and animal models that cover at least genotypes A to E and represent the vast majority of global HBV infections to test novel treatment strategies.

## 1. Introduction

An estimated 250 million humans [1] are chronically infected with hepatitis B virus (HBV), causing an estimated 887,000 annual deaths, mostly due to the long-term sequelae liver cirrhosis and hepatocellular carcinoma (HCC) [2]. The viral population can be divided into nine genotypes (A to I) [3,4] which differ in more than 7.5% of their nucleotide sequences [3,4] and which are further subdivided into subgenotypes with a nucleotide divergence greater than 4% [3,4]. While genotypes A to H have long been accepted as individual genotypes, two new genotypes (I and J) were proposed more recently [5,6]. Genotype I was first described in 2008 after isolation from a Vietnamese patient and constitutes a recombination of genotypes A, C and G [5]. Since the nucleotide divergence, especially compared to genotype C, is relatively small, it was long debated if this strain should be considered a new genotype [7]. Finally, the identification of similar HBV strains in Laos, North India and China in isolated native populations, indicating that the virus strains have circulated in these populations for a longer time, led to acceptance as an independent genotype [8,9,10]. Another strain, previously proposed to be a new genotype “J”, was isolated from a Japanese patient who had lived on Borneo island [6]. Phylogenetic analysis revealed that the strain rather resembles gibbon than human HBV, and may result from recombinations with human genotype C [11]. Since further reports of human infections with this strain are lacking, it has so far not been recognized as a relevant HBV genotype [11]. 

Sequencing and genotyping of HBV isolates is not routinely done and rarely reported in, e.g., epidemiological studies. Hepatitis B virus genotypes, however, vary in their clinical consequences including the natural course of infection, disease progression and treatment response (reviewed in [3,12,13]): While genotypes B, C and I are associated with a more frequent vertical transmission from mother to child, a higher transmission rate during sexual contact or injecting drug use has been reported for genotypes A, D and G [3,14,15,16]. A higher chronification rate after infections with genotypes A and C, compared to genotypes B and D, has also been reported [14], but may also be due to the transmission route. Among chronic HBV carriers, a lower rate of seroconversion to HBV-e-antigen antibodies (anti-HBe) was proposed in genotype C and D infections [14]. Also, a faster disease progressions to liver cirrhosis and HCC are associated with infections with genotypes C, D and F [14]. While all genotypes similarly respond to treatment with reverse transcriptase inhibitors, under interferon-α treatment, genotypes A and B show an increased virological response and higher anti-HBe seroconversion than other HBV genotypes [14,17].

The separation of the HBV population into different genotypes can be dated back 30 million years ago, when the ancestors of modern *Homo sapiens* dispersed across Africa and Eurasia [18]. The distinct appearances of genotypes and subgenotypes in certain geographical regions and ethnic groups [3,4,12] allow for the confirmation of prehistoric human migrations by the transfer of HBV genotypes and subtypes from one continent to another [3,19]. While previous studies described differences between local genotype appearances, the number of infections with each HBV genotype and the genotype distribution within global chronic HBV infections have not been studied. Given the specific characteristics of each HBV genotype, this information could, however, be valuable for precising the HBV disease burden and informing health policy, for example, by means of predicting and estimating prevention and treatment needs and relevance of specific treatments in a country. A further adaptation and rational application of diagnostic tests, as well as the development of new broadly applicable therapies, may also be stimulated by scoping HBV genotypes worldwide. We approached this need and performed a literature search for HBV genotyping studies and applied previously published HBV surface antigen (HBsAg) prevalence estimates [1] and United Nations (UN) population data [20] to approximate the number of HBV infections by each genotype per country, world region and globally.

## 2. Materials and Methods

### 2.1. Literature Search for Hepatitis B Virus Genotyping Data

**Figure 1:**
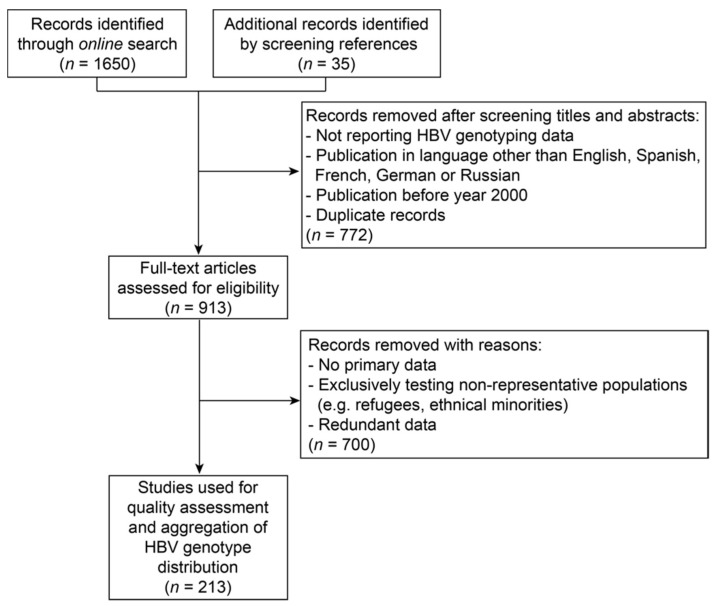
Flow chart on selection of records describing primary hepatitis B virus (HBV) genotyping data.

A literature search was performed up to 23^rd^ January 2018 using Google Scholar and the search terms: [Country Name] and [“HBV” or “Hepatitis B Virus”] and [“genotype”]. Countries entered were these for which HBsAg prevalence data were reported by Schweitzer et al. [1]. If no publications were found for a certain country, the search was repeated with names of major cities within the respective country instead of the country name. Additional publications were added after manually screening references in identified records. Publications were subjected to a two-step review. First, titles and abstracts in English, Spanish, French, German or Russian languages were screened with regard to the provision of HBV genotyping results. If a manuscript was not accessible, data were extracted from its abstract if possible or taken from a secondary publication. Publications before the year 2000 and duplicate records were excluded (Figure 1). 

Other exclusion criteria applied during full text screening were:

- Study population was based exclusively on individuals of foreign origin (e.g., refugees) or an ethnical minority. If information on country of origin was available, data were allocated to the respective country;
- Secondary data;
- Redundant data which were also described in another record.

### 2.2. Extracted Variables and Assumptions

From manuscripts, which were deemed relevant during the full text review, the following information was extracted (for variable characteristics, see Table S1 in Supplementary Materials):

- Year of publication;
- Publication type;
- Year of sample collection (if no year was available, two years prior to publication was assumed);
- Date of analysis (if no information was available, one year prior to publication was assumed);
- Location of sample collection;
- Selection criteria of study participants;
- Sex and age of tested population;
- Method of genotyping;
- Number of samples and result of genotyping.

### 2.3. Qualitative Assessment of Data

A scoring system was developed for a descriptive assessment of the quality of genotyping data that were deemed relevant during full text screening.

1. A study quality score was calculated with equal weighting from two different scores, the genotyping and the generalizability score (lowest score implying the lowest quality):
  (a) Genotyping score: Reliability of genotype information. A weighted average was calculated from scores for:
    ○ Year of sample analysis in studies (30% weighting) (median was used in case of year range provided):
      ▪ Before 2010 (before genotype I was described): Score 1;
      ▪ 2010 or later: Score 2.
    ○ Ability of the method to correctly identify genotype, including recombinant viruses (e.g., genotype I) (70% weighting):
      ▪ Non-sequencing-based methods like probe-/PCR-/restriction fragment length polymorphism (RFLP), or enzyme immunoassay (EIA) (partly not capable to detect all genotypes/high risk to misclassify recombinant viruses): Score 1;
      ▪ Region of viral genome sequenced (capable to identify all genotypes but with medium risk to misclassify recombinant viruses): Score 3;
      ▪ Whole viral genome sequenced (capable to identify all genotypes including recombinant viruses): Score 5.
  (b) Generalizability score: Potential for generalizability of genotype information to chronic HBV infections in a country in the year 2015. A weighted average was calculated from scores for:
    ○ Representation of country by the study location (40% weighting):
      ▪ Samples derived from a single town or region: Score 1;
      ▪ Samples collected from several regions or nationwide: Score 2.
    ○ Representation of HBV-infected population by the study population (40% weighting):
      ▪ Favored selection (e.g., individuals of specific age group, with a specific risk factor to acquire HBV infection or showing a specific sequala): Score 1;
      ▪ Non-favored selection (e.g., all HBV-DNA positive individuals): Score 2.
    ○ Year of sample collection (20% weighting). Median was used in case of year range provided:
      ▪ Before 2000: Score 1;
      ▪ 2000–2010: Score 2;
      ▪ 2011 or later: Score 3.
2. A country quality score was calculated as the weighted average of study quality scores of all studies providing results for a certain country. Each study was weighted by the proportion of samples it contributed. The result was multiplied by a score for the sum of genotyped samples from all studies for the country:
  ▪ <100 samples: Score 1;
  ▪ 100–999 samples: Score 2;
  ▪ 1000 or more samples: Score 3.

The resulting score was fitted to a 1–10 scale.

### 2.4. Aggregation of Genotyping Data

If several sources provided genotyping results for a country, data from studies were aggregated by weighting each study by the proportion of genotyped samples it contributed. The other quality parameters were not included at this step. Inter-genotype recombinant viruses were not allocated to a specific genotype but reported separately together with co-infections with more than one genotype and samples, from which the genotype could not be determined (Table S1, Supplementary Materials). 

### 2.5. Approximation of Number of Infections with Each Hepatitis B Virus Genotype

Prevalence of HBsAg as a marker of chronic HBV infection was retrieved from Schweitzer et al. [1]. Population data were extracted from the United Nations World Population Prospect (UN WPP) [20] for the year 2015 to calculate the number of HBV infections per country. To approximate the number of infections with a certain genotype, genotype frequency was multiplied by the number of HBV infections for each country. Data aggregation was performed in an automated way using custom coded scripts.

### 2.6. Creation of Maps and Additional Software

Maps were created using the Quantum Geographic Information System (QGIS) (http://www.qgis.org/en/site/) with the map file World Borders Dataset from Thematic Mapping (http://thematicmapping.org/downloads/world_borders.php) and the plugin MMQGIS (https://plugins.qgis.org/plugins/mmqgis/). The quality score was calculated in Microsoft Excel for Mac Version 16.13.1. Diagrams were created with Prism 6.0f for Mac. Custom scripts were coded in the Ruby programming language.

## 3. Results

### 3.1. Description of Hepatitis B Virus Genotyping Data

One thousand, six hundred and fifty records were identified through online search, and 35 additionally by screening references in identified records. Studies reporting HBV genotyping data, which were published between 1 January 2000 and 23 January 2018, were subjected to a selection process, as described in Figure 1 and ‘Materials and Methods’.

**Figure 2:**
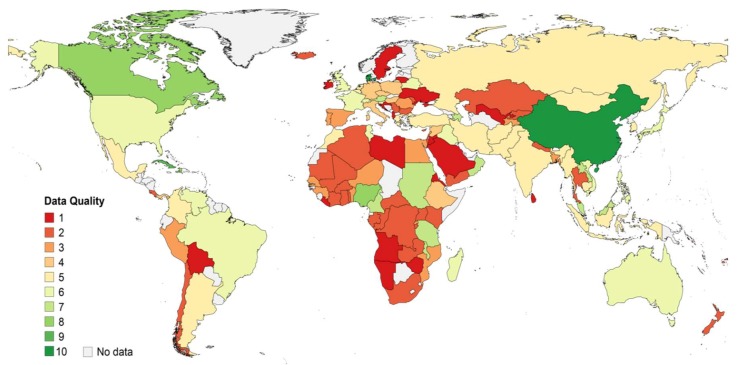
Quality of acquired HBV genotyping data per country. One (red) indicates the lowest and ten (green) represents the highest data quality. The scoring system was based on the sampling region, study population, sampling year, genotyping method, year of analysis, and number of analyzed samples.

Two hundred and thirteen publications were included in the data aggregation (Table S1, File S1, Supplementary Materials), composed of 95% peer-reviewed and 5% non-peer-reviewed (e.g., posters) publications. The majority of samples were collected between the years 1996 to 2015, with oldest samples derived from 1984 (Figure S1A, Supplementary Materials). Publication dates were equally distributed across years 2000 to 2018 (Figure S1B, Supplementary Materials). In 62% of studies, the sampling region covered small towns or areas of a country, whereas 38% of studies collected samples from several regions or nationwide. The majority (75%) of studies sequenced the viral genome to determine the genotype, with 18% of studies sequencing the whole and 57% parts of the genome. Twenty-five percent of studies performed alternative, mostly probe- or PCR-based assays to determine the genotype. Overall, included records encompass genotyping results from 26,319 HBV-infected individuals from 125 countries. The availability and quality of data varied between countries (Table S1, Supplementary Materials, and Figure 2). According to the scoring system applied in this study, most relevant data were available for Denmark, China and Cuba, for which in recent years, large numbers of samples from HBV-infected individuals from several regions of the respective country were sequenced. In contrast, quality of data was assessed to be lowest for records from Sweden, Namibia, Ireland, Angola, Bolivia, Eritrea, Liberia and Sri Lanka. This was mostly due to a small number of samples derived from a single study site, which were analyzed using non-sequencing-based methods. When relating to the number of HBV infections, about 1/3^rd^ of total HBV infections were represented by low (Scores 1–3), medium (Scores 4–7) or high (Scores 8–10) quality genotyping data. Quality was homogenously distributed across genotypes but rated best for genotypes B, C and I, mainly because of their frequent appearance in China for which good quality data were available (Figure S1C,D, Supplementary Materials).

### 3.2. World-Wide Hepatitis B Virus Genotype Appearance

**Figure 3:**
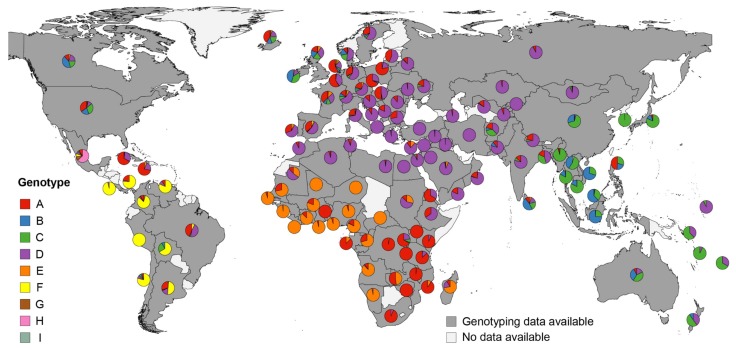
Distribution of HBV genotypes by country. Pie charts indicate proportional HBV genotype distributions in the respective countries. Genotype distributions within samples with successful genotyping are presented, excluding inter-genotype recombinant viruses, co-infections with more than one HBV genotype or undefined infections. Underlying literature sources and number of sequenced isolates are given in Table S1 (Supplementary Materials).

In most European countries, genotypes A and D were found to be most common, with an increasing appearance of genotype A towards Northwestern Europe (Figure 3). In Eastern Europe and Western-, Central-, North- and South Asia as well as Northern Africa, genotype D was predominant without further genotypes present in a significant number. On the British Isles and Denmark, genotypes B and C were also common. In Eastern and Southeastern Asia, as well as Australasia and Oceania, genotype C was the most frequent genotype. Besides genotype C, genotype B also constituted a relative high proportion in China, Southeast Asia, and Australia, whereas genotype D was more frequent in Australasia and Oceania. One exception was the Philippines, where genotype A constituted about half of infections. In Sub-Saharan Africa (comprising Eastern Africa, Middle Africa, Southern Africa, and Western Africa), a distinct genotype distribution was found, with genotype E predominating in the western part, and decreasing proportions towards Eastern Africa, where, with the exception of Madagascar (genotype E), mainly genotype A was found. In Latin America, three genotypes (F, G and H) were found that are rare in other parts of the world. Genotype F was predominant in most Latin American countries, for which HBV sequencing data were identified. Brazil and Mexico differed from the other countries in the region. In Brazil, genotypes A and D dominated, and uniquely in Mexico, genotypes G (10.2%) and H (63.3%) were found in relevant numbers. Northern America and the Caribbean differed in their genotype distribution from Latin America. In Northern America, genotype A, B, C and D were predominant, whereas in the Caribbean, mainly genotype A, and, to a lesser extent, genotype D were found (for details, see Table S1, Supplementary Materials). Taken together, the HBV genotype distribution showed similar patterns between countries of the same world region but strongly varied between different parts of the world.

### 3.3. Approximation of Number of Chronic Hepatitis B Virus Infections with Each Hepatitis B Virus Genotype

**Figure 4:**
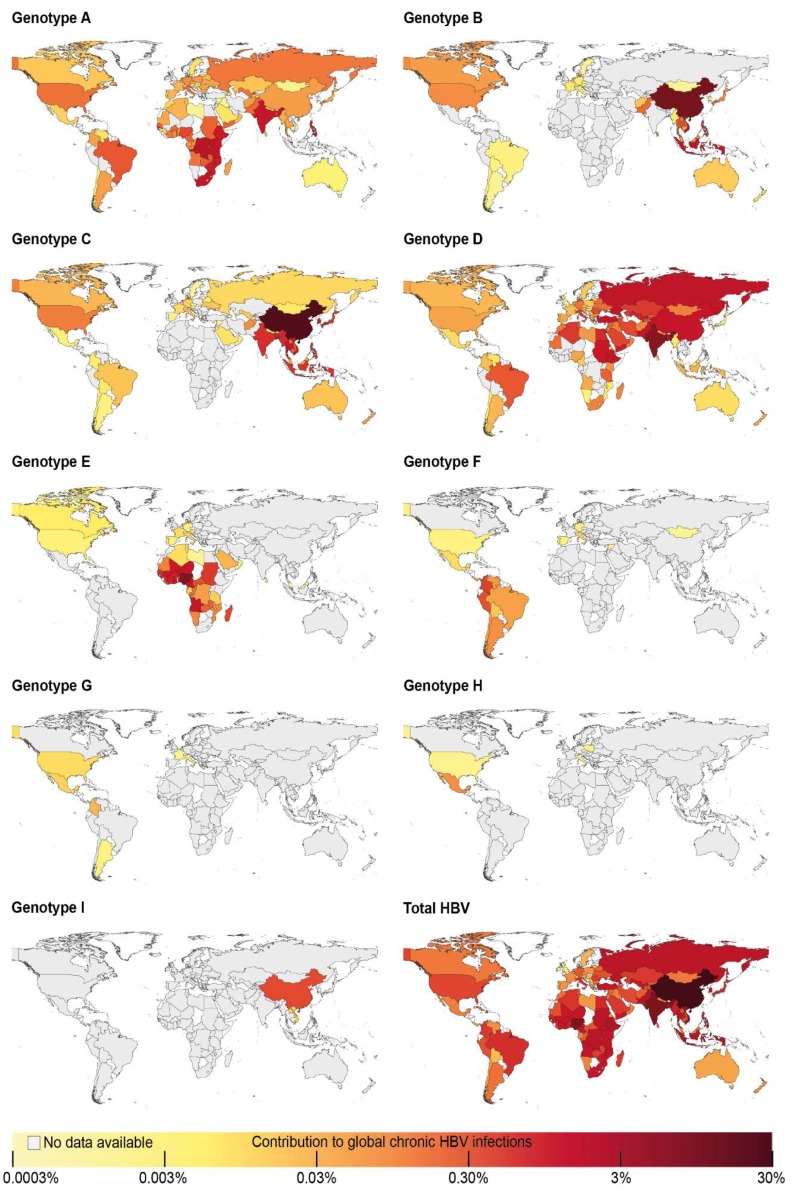
Contribution of genotypes to global chronic HBV infections. The number of infections with each genotype in a respective country is illustrated as percentage of global chronic HBV infections.

**Figure 5:**
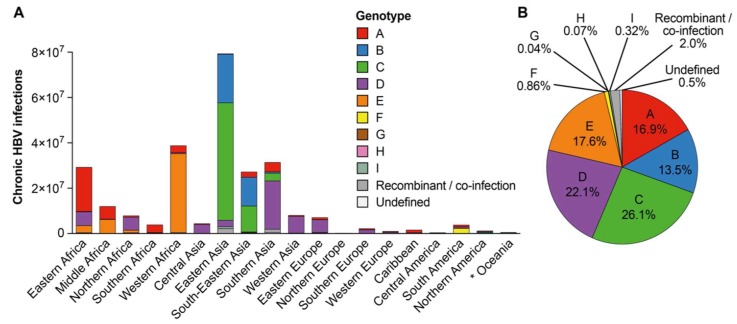
Approximation of the contribution of HBV genotypes to global burden of chronic HBV infection. (**A**) Estimation of the number of chronic infections with each genotype per world region. *: Oceania includes pooled data of Australia/New Zealand, Melanesia, Micronesia, and Polynesia. (**B**) Approximation of the genotype distribution within global chronic HBV infections. Values <2% are given with two decimals to prevent distortion of genotype distribution. Recombinant/co-infection: infection with an inter-genotype recombinant or with more than one HBV genotype; Undefined: genotype allocation not possible.

We approximated the number of infections with a certain HBV genotype by extrapolating the genotype distribution found in study populations to all HBV-infected individuals in a respective country, using HBsAg prevalence estimates by Schweitzer et al. [1] and UN Population data for the year 2015 [20] (Table S2, Supplementary Materials). The distinct differences between the number of HBV-infected individuals in different world regions, together with variations in genotype frequencies, determined the total number of infections with each genotype in countries (Figure 4; Table S3, Supplementary Materials): This was most apparent for genotypes with high frequencies in regions harboring large HBV-infected populations, such as Sub-Saharan Africa (32.3% of global HBV infections, mostly genotypes A and E) or Eastern and Southeast Asia (together 36.6% of global HBV infections, mostly genotypes B and C) (Figure 4 and Figure 5A; Table S3, Supplementary Materials). Overall, genotype C infected 26.1% of HBsAg-positive individuals worldwide and thus caused the highest number of HBV infections (Figure 4 and Figure 5B), with 98.6% of genotype C infections occurring in Asia (Table S3, Supplementary Materials). Genotype D was found in 22.1% of all HBV-infected individuals, of which 61.9% were found in Asia with 22.0% in Africa and 13.5% in Europe. Genotype E caused 17.6% of all HBV infections globally, of which 97.0% occurred in Sub-Saharan Africa. Genotype A caused 16.9% of HBV infections worldwide, of which a majority of 72.2% was found in Sub-Saharan Africa followed by 17.2% in Asia. Globally, 13.5% of chronic HBV infections were caused by genotype B, of which 98.8% occurred in Asia. In summary, genotypes A to E were estimated to together cause 96.2% of global chronic HBV infections. The other 4 genotypes F to I together accounted only for 1.3% of all infections and occurred mostly in Latin America (genotypes F to H) or Eastern Asia (genotype I); 2.5% of global infections were described as infections with inter-genotype recombinants or mixed infections with more than one genotype or could not be defined (Figure 5B).

## 4. Discussion

Information on the contributions of each HBV genotype to the global burden of HBV infection is missing. This scoping review on HBV genotyping results and the combination with HBsAg prevalence and population estimates approaches this gap. We found that the different distributions of HBV genotypes, together with varying numbers of chronically HBV-infected individuals in world regions, result in strong variations in the global number of infections caused by each genotype: genotypes A to E were estimated to cause the vast majority of infections, although each in different parts of the world accounting together for 96% of chronic HBV infections worldwide. In contrast, the remaining genotypes F to I each were estimated to cause a significantly lower number of infections, together causing less than 2% of chronic HBV infections in the world.

The number and quality of data identified by our scoping review strongly varied between countries even within highly developed regions of the world. In each world region, there were countries with good, but also with poor quality data or no data at all. The distribution of HBV genotypes showed an interrelation with geographic boundaries (e.g., oceans or the Sahara Desert) or the dissemination of ethnic groups. For instances, while HBV-infections in Sub-Saharan Africa were primarily caused by genotypes A and E, this was not the case in Northern Africa. Here, the population, which rather resembles the populations of West Asia [21], showed also a similar HBV genotype distribution as this region (mainly genotype D). Additionally, world areas, in which large proportions of the population descend from migrants from other parts of the world, showed a genotype distribution reflecting the areas, from which migrants originated. This is illustrated by, for example, relative high frequencies of genotypes A, B, C and D in Northern America referring to migrants from Europe and Asia. Another example constitutes the Caribbean, where mostly genotypes A and D were found, correlating with higher proportions of migrants originating from the African continent. As a consequence of different HBV endemicity levels and population sizes, genotypes with a high occurrence in regions, such as Southeast and East Asia (genotypes B and C) and Sub-Saharan Africa (genotypes A and E), caused a large proportion of worldwide infections. In contrast, genotype F, which was dominant in Latin America, was estimated to cause less than 1% of chronic HBV infections globally, due to a relatively low HBV endemicity in this area.

Our results need to be interpreted with caution, as several technical limitations are inherent in the underlying data and the method of extrapolating the genotype distribution from study populations to global HBV infections. A potential cause of bias is the genotyping method applied: the majority of studies only sequenced parts of the viral genome or used non-sequencing-based methods. This can lead to wrong classifications, especially for recombinant viruses (including genotype I which constitutes a recombination of genotype A, C and G) [22]. In some instances, tests were used (e.g., line probe assays) with inability to detect all genotypes. Another impacting factor is the analysis year of samples: almost half of studies were published before 2010, i.e., before genotype I was described or recognized as an independent genotype. While current data confirm that genotype I is rare and only found in Southeast Asia, there is a potential that we underestimated its frequency, because infections with genotype I were missed in earlier studies. 

Regarding the precision of extrapolating the genotype distribution found in a study population to all HBV-infected individuals of the respective country, some limitations need to be mentioned. Two-thirds of studies were based on a single town or region in a country. This region may not reflect HBV-infected individuals in the whole country, especially in cases, where ethnic groups that potentially carry different HBV genotypes live in separate geographic areas. However, a significant number of studies which sampled more than one region did not do this in a manner that would represent the whole country in a satisfying way, which was especially a problem for large countries (e.g., Russia). 

In 40% of studies included, we identified a risk to potentially favor selection of certain genotypes. This, for example, applies to studies which exclusively included patients with advanced fibrosis/cirrhosis or HCC which could favor the selection of genotypes that are associated with a faster disease progression. Additionally, often genotypes were not detected at all in included studies for a country, but studies testing specific minorities or non-national immigrants in the country (which we excluded due to our exclusion criteria) proofed the presence of these genotypes at least at low level. This suggests that rare genotypes were often not detected, most likely because of small sample sizes or because certain minorities were not represented in study populations. 

Low numbers of genotyped samples could also result in an imprecise estimation of the genotype distribution in several countries. For many countries, the sample size could be enlarged by pooling samples from several studies, but this was not possible for all countries. Further compromising the precision of the global genotype distribution, the other parameters used—HBsAg prevalence and population data—also only constitute estimates and in most cases did not derive from the same time point and might have changed over time. Moreover, some genotyping studies included samples which were either collected several years ago or during a large period, questioning representativeness for the year 2015. We furthermore could only calculate the number of genotype infections if all three parameters were available for a country. While we were able to retrieve data for 125 countries which, according to the UN WPP, represented 96% of the world population in 2015, we could not calculate genotype infections for the remaining countries. Future studies should focus on countries with highest need to perform HBV genotyping, including countries for which no data could be retrieved or countries for which we assessed available data to have poor quality (Table S1, Supplementary Materials). 

Importantly, while we assessed the quality of studies using a scoring system, this served only descriptive purposes. Due to limitation of data, we did not adjust for factors besides the sample number in our aggregation analyses. We also did not include age as a factor which is of note due to the interrelation of transmission routes and genotypes which could lead to varying genotype frequencies between age groups. We omitted these factors from our analysis, as for many countries, only a single source was available and information on the age of individuals infected with a certain genotype was mostly missing. The scarcity of genotyping data, when compared to seroprevalence studies, probably results from the fact that genotyping, at least when performed by sequencing, is labor and cost-intensive, which constitutes a limitation, especially for resource-poor countries. However, we also cannot exclude that available genotyping data were missed by our literature search. As a consequence of the scarcity of HBV genotyping data, the extrapolation of genotype distribution was based on only 26,000 genotyped samples, which constitutes only around 0.01% of chronic HBV infections. Thus, the global HBV genotype distribution calculated in our study carries the risk of high uncertainties, which we did not define due to limited data points. The global genotype distribution calculated in this study should, therefore, be regarded as a first approximation, but a more precise estimation is warranted, by taking other factors and co-variates into consideration and by including additional data that may come up.

We chose not to extend our study to include subgenotypes for several reasons: (i) Only few publications included information on subgenotypes; (ii) the majority of studies used genotyping methods, which are not suitable to determine subgenotypes, including sequencing of only parts of the viral genome [4]; and (iii) the definition of subgenotypes has been subject to changes during the phase, from which genotyping studies were selected [4,23,24,25]. Thus, we believe the quantity and quality of available data were not sufficient to allow estimation of the subgenotype distribution with sufficient precision.

Despite the limitations described, this study provides an up-to-date insight into the worldwide HBV genotyping data from recent years and is an important initial approach to quantifying how genotypes contribute to the global burden of chronic HBV infection. Our study identified countries with no or only low quality genotyping data urgently requiring further studies. The wide distribution of HBV genotypes around the world underscores the need to ensure that the applied diagnostic tests and therapeutic approaches address the variety of HBV genotypes. Most experimental cell culture and animal models currently used to study HBV biology and new treatment options are based on genotype D or A, which we estimated, account for only 1/5th and 1/6th of infections worldwide, respectively. This reflects the need to expand experimental models to the other HBV genotypes. While efforts are ongoing to establish models for genotypes B and C, genotype E seems to be disregarded in this respect, although it seems more important than genotypes A and B. Experimental models for drug development should be expanded to at least cover genotypes A to E to represent the vast majority of HBV infections worldwide.

## Supplementary Materials

The following are supplied with the manuscript and are available online at http://www.mdpi.com/2073-4425/9/10/495/s1: Table S1: Results from literature review for HBV genotyping data including quality score File S1: List of references from which genotyping data was extracted; Figure S1: Quality of included genotyping data; Table S1: Results from literature review for HBV genotyping data including the quality score; Table S2: Extrapolation of genotype distribution to all HBV-infected individuals of the country; Table S3. Geographical dispersal of HBV infections and genotypes.
