# Supplementary material for: The Global Hepatitis B Virus Genotype Distribution Approximated from Available Genotyping Data
Source: Genes (Basel). 2018 Oct 15;9(10):495. doi: 10.3390/genes9100495 (PMC6210291; doi:10.3390/genes9100495)
Supplement: Supplementary file 1 [file genes-09-00495-s001.zip › Supplementary files_new/File S1.docx]

File S1. List of references from which genotyping data was extracted

1. Attaullah, S., Ur Rehman, S., Khan, S., Ali, I., Ali, S., and Khan, S. N. "Prevalence of Hepatitis B Virus Genotypes in Hbsag Positive Individuals of Afghanistan." *Virol J* 8, no. 1 (2011): 281.

2. Kondili, L. A., Brunetto, M. R., Maina, A. M., Argentini, C., Chionne, P., La Sorsa, V., Resuli, B., Mele, A., and Rapicetta, M. "Clinical and Molecular Characterization of Chronic Hepatitis B in Albania: A Country That Is Still Highly Endemic for Hbv Infection." *J Med Virol* 75, no. 1 (2005): 20-6.

3. Ezzikouri, S., Pineau, P., and Benjelloun, S. "Hepatitis B Virus in the Maghreb Region: From Epidemiology to Prospective Research." *Liver Int* 33, no. 6 (2013): 811-9.

4. Valente, F., Lago, B. V., Castro, C. A., Almeida, A. J., Gomes, S. A., and Soares, C. C. "Epidemiology and Molecular Characterization of Hepatitis B Virus in Luanda, Angola." *Mem Inst Oswaldo Cruz* 105, no. 8 (2010): 970-7.

5. Barbini, L., Elizalde, M., Torres, C., and Campos, R. "Molecular Epidemiology and Genetic Diversity of Hepatitis B Virus in Mar Del Plata City, Argentina." *Infect Genet Evol* 19 (2013): 152-63.

6. Pezzano, S. C., Torres, C., Fainboim, H. A., Bouzas, M. B., Schroder, T., Giuliano, S. F., Paz, S., Alvarez, E., Campos, R. H., and Mbayed, V. A. "Hepatitis B Virus in Buenos Aires, Argentina: Genotypes, Virological Characteristics and Clinical Outcomes." *Clin Microbiol Infect* 17, no. 2 (2011): 223-31.

7. Trinks, J., Frias, S., Frider, B., Alessio, A., Pozzati, M., Daleoso, G., Leon, L., Batalla, V. M., Diaz, A., Ameigeiras, B., and Oubina, J. R. "Genotypes B and C Hepatocellular Carcinoma-Associated Hepatitis B Virus Pre-S Mutants: Their Detection among F1b and A2 - but Not F4 - Isolates from Argentina." *J Viral Hepat* 19, no. 11 (2012): 823-8.

8. Bertolini, D. A., Gomes-Gouvea, M. S., Guedes De Carvalho-Mello, I. M., Saraceni, C. P., Sitnik, R., Grazziotin, F. G., Laurino, J. P., Fagundes, N. J., Carrilho, F. J., and Pinho, J. R. "Hepatitis B Virus Genotypes from European Origin Explains the High Endemicity Found in Some Areas from Southern Brazil." *Infect Genet Evol* 12, no. 6 (2012): 1295-304.

9. Mandorfer, M. "P1293 Epidemiology of Chronic Hepatitis B in Austria." *J Hepatol* 60, no. 1 (2014): S521.

10. Ratnam, D., Dev, A., Nguyen, T., Sundararajan, V., Harley, H., Cheng, W., Lee, A., Rusli, F., Chen, R., and Bell, S. "Efficacy and Tolerability of Pegylated Interferon‐Α‐2a in Chronic Hepatitis B: A Multicenter Clinical Experience." *J Gastroenterol Hepatol* 27, no. 9 (2012): 1447-53.

11. Bokharaei-Salim, F., Keyvani, H., Monavari, S. H., Esghaei, M., Fakhim, S., Ataei Pirkooh, A., and Behnava, B. "Distribution of Hepatitis B Virus Genotypes in Azerbaijani Patients with Chronic Hepatitis B Infection." *Hepat Mon* 14, no. 12 (2014): e25105.

12. Mohammadnejad, L., Farajnia, S., Parivar, K., Naghili, B., and Yousefzadeh Kheirnagsh, R. "Hepatitis B Virus Genotypes in Eastern Azerbaijan, Northwest Iran." *Arch Iran Med* 15, no. 7 (2012): 446-8.

13. Shaha, M., Hoque, S. A., and Rahman, S. R. "Molecular Epidemiology of Hepatitis B Virus Isolated from Bangladesh." *Springerplus* 5, no. 1 (2016): 1513.

14. Munshi, S. U., Tran, T. T. T., Vo, T. N. T., Tabassum, S., Sultana, N., Nguyen, T. H., Jahan, M., Le, C. N., Baker, S., and Rahman, M. "Molecular Characterization of Hepatitis B Virus in Bangladesh Reveals a Highly Recombinant Population." *PLoS One* 12, no. 12 (2017): e0188944.

15. Gasich, E., Eremin, V., Sasinovich, S., and Tulinova, M. "Hbv and Hcv Genotypes Distribution on the Territory of Belarus." *Retrovirology* 9, no. 1 (2012): P57.

16. Olinger, C., Lazouskaya, N., Eremin, V., and Muller, C. "Multiple Genotypes and Subtypes of Hepatitis B and C Viruses in Belarus: Similarities with Russia and Western European Influences." *Clinical Microbiology and Infection* 14, no. 6 (2008): 575-81.

17. Micalessi, M. I., De Cock, L., and Vranckx, R. "Hepatitis B Virus (Hbv) Genotyping in Belgian Patients with Chronic Hbv Infection." *Clin Microbiol Infect* 11, no. 6 (2005): 499-501.

18. Fujiwara, K., Tanaka, Y., Orito, E., Ohno, T., Kato, T., Sugihara, K., Hasegawa, I., Sakurai, M., Ito, K., Ozasa, A., Sakamoto, Y., Arita, I., El-Gohary, A., Benoit, A., Ogoundele-Akplogan, S. I., Yoshihara, N., Ueda, R., and Mizokami, M. "Distribution of Hbv Genotypes among Hbv Carriers in Benin:Phylogenetic Analysis and Virological Characteristics of Hbv Genotype E." *World J Gastroenterol* 11, no. 41 (2005): 6410-5.

19. Khan, A., Tanaka, Y., Saito, H., Ebinuma, H., Sekiguchi, H., Iwama, H., Wakabayashi, G., Kamiya, T., Kurbanov, F., Elkady, A., and Mizokami, M. "Transmission of Hepatitis B Virus (Hbv) Genotypes among Japanese Immigrants and Natives in Bolivia." *Virus Res* 132, no. 1-2 (2008): 174-80.

20. Castilho Mda, C., Oliveira, C. M., Gimaque, J. B., Leao, J. D., and Braga, W. S. "Epidemiology and Molecular Characterization of Hepatitis B Virus Infection in Isolated Villages in the Western Brazilian Amazon." *Am J Trop Med Hyg* 87, no. 4 (2012): 768-74.

21. Viana, S., Paraná, R., Moreira, R. C., Compri, A. P., and Macedo, V. "High Prevalence of Hepatitis B Virus and Hepatitis D Virus in the Western Brazilian Amazon." *Am J Trop Med Hyg* 73, no. 4 (2005): 808-14.

22. Eloy, A. M., Moreira, R. C., Lemos, M. F., Silva, J. L., and Coelho, M. R. "Hepatitis B Virus in the State of Alagoas, Brazil: Genotypes Characterization and Mutations of the Precore and Basal Core Promoter Regions." *Braz J Infect Dis* 17, no. 6 (2013): 704-6.

23. Sitnik, R., Pinho, J. R., Bertolini, D. A., Bernardini, A. P., Da Silva, L. C., and Carrilho, F. J. "Hepatitis B Virus Genotypes and Precore and Core Mutants in Brazilian Patients." *J Clin Microbiol* 42, no. 6 (2004): 2455-60.

24. Chacha, S. G. F., Gomes-Gouvea, M. S., Malta, F. M., Ferreira, S. D. C., Villanova, M. G., Souza, F. F., Teixeira, A. C., Passos, A., Pinho, J. R. R., and Martinelli, A. L. C. "Distribution of Hbv Subgenotypes in Ribeirao Preto, Southeastern Brazil: A Region with History of Intense Italian Immigration." *Braz J Infect Dis* 21, no. 4 (2017): 424-32.

25. Barros, L. M. F., Gomes-Gouvêa, M. S., Kramvis, A., Mendes-Corrêa, M. C. J., Dos Santos, A., Souza, L. a. B., Santos, M. D. C., Carrilho, F. J., De Jesus Domicini, A., and Pinho, J. R. R. "High Prevalence of Hepatitis B Virus Subgenotypes A1 and D4 in Maranhão State, Northeast Brazil." *Infection, genetics and Evolution* 24 (2014): 68-75.

26. Oliveira, M. P., Lemes, P. S., Matos, M. a. D., Del‐Rios, N. H. A., Carneiro, S., Aparecida, M., Silva, C., Macedo, Á., Lopes, C. L. R., and Teles, S. A. "Overt and Occult Hepatitis B Virus Infection among Treatment‐Naïve Hiv‐Infected Patients in Brazil." *Journal of medical virology* 88, no. 7 (2016): 1222-29.

27. Ciccozzi, M., Babakir‐Mina, M., Lo Presti, A., Salpini, R., Cella, E., Gabanelli, E., Teoharov, P., Kevorkyan, A., Perno, C. F., and Zehender, G. "Molecular Analysis of Hepatitis B Virus in Bulgaria." *Journal of medical virology* 85, no. 1 (2013): 49-54.

28. Diarra, B., Yonli, A. T., Sorgho, P. A., Compaore, T. R., Ouattara, A. K., Zongo, W. A., Tao, I., Traore, L., Soubeiga, S. T., Djigma, F. W., Obiri-Yeboah, D., Nagalo, B. M., Pietra, V., Sanogo, R., and Simpore, J. "Occult Hepatitis B Virus Infection and Associated Genotypes among Hbsag-Negative Subjects in Burkina Faso." *Mediterr J Hematol Infect Dis* 10, no. 1 (2018): e2018007.

29. Ntagirabiri, R., Munezero, B., Nahimana, C., and Ndabaneze, E. "[Hepatitis B Virus Genotypes and Evolutionary Markers in Chronic Hbsag Patients in Bujumbura]." *Pan Afr Med J* 23 (2016): 95.

30. Huy, T. T., Sall, A. A., Reynes, J. M., and Abe, K. "Complete Genomic Sequence and Phylogenetic Relatedness of Hepatitis B Virus Isolates in Cambodia." *Virus Genes* 36, no. 2 (2008): 299-305.

31. Srey, C. T., Ijaz, S., Tedder, R. S., and Monchy, D. "Characterization of Hepatitis B Surface Antigen Strains Circulating in the Kingdom of Cambodia." *J Viral Hepat* 13, no. 1 (2006): 62-6.

32. Sa-Nguanmoo, P., Tangkijvanich, P., Thawornsuk, N., Vichaiwattana, P., Prianantathavorn, K., Theamboonlers, A., Tanaka, Y., and Poovorawan, Y. "Molecular Epidemiological Study of Hepatitis B Virus among Migrant Workers from Cambodia, Laos, and Myanmar to Thailand." *J Med Virol* 82, no. 8 (2010): 1341-9.

33. Forbi, J. C., Ben-Ayed, Y., Xia, G. L., Vaughan, G., Drobeniuc, J., Switzer, W. M., and Khudyakov, Y. E. "Disparate Distribution of Hepatitis B Virus Genotypes in Four Sub-Saharan African Countries." *J Clin Virol* 58, no. 1 (2013): 59-66.

34. Mulders, M. N., Venard, V., Njayou, M., Edorh, A. P., Bola Oyefolu, A. O., Kehinde, M. O., Muyembe Tamfum, J. J., Nebie, Y. K., Maiga, I., Ammerlaan, W., Fack, F., Omilabu, S. A., Le Faou, A., and Muller, C. P. "Low Genetic Diversity Despite Hyperendemicity of Hepatitis B Virus Genotype E Throughout West Africa." *J Infect Dis* 190, no. 2 (2004): 400-8.

35. Singh, A. E., Plitt, S. S., Osiowy, C., Surynicz, K., Kouadjo, E., Preiksaitis, J., and Lee, B. "Factors Associated with Vaccine Failure and Vertical Transmission of Hepatitis B among a Cohort of Canadian Mothers and Infants." *J Viral Hepat* 18, no. 7 (2011): 468-73.

36. Panessa, C., Hill, W. D., Giles, E., Yu, A., Harvard, S., Butt, G., Andonov, A., Krajden, M., and Osiowy, C. "Genotype D Amongst Injection Drug Users with Acute Hepatitis B Virus Infection in British Columbia." *J Viral Hepat* 16, no. 1 (2009): 64-73.

37. Huynh, C., Minuk, G. Y., Uhanova, J., Baikie, M., Wong, T., and Osiowy, C. "Serological and Molecular Epidemiological Outcomes after Two Decades of Universal Infant Hepatitis B Virus (Hbv) Vaccination in Nunavut, Canada." *Vaccine* 35, no. 35 Pt B (2017): 4515-22.

38. Osiowy, C., Larke, B., and Giles, E. "Distinct Geographical and Demographic Distribution of Hepatitis B Virus Genotypes in the Canadian Arctic as Revealed through an Extensive Molecular Epidemiological Survey." *J Viral Hepat* 18, no. 4 (2011).

39. Congly, S. E., Wong, P., Al-Busafi, S. A., Doucette, K., Fung, S. K., Ghali, P., Fonseca, K., Myers, R. P., Osiowy, C., and Coffin, C. S. "Characterization of Hepatitis B Virus Genotypes and Quantitative Hepatitis B Surface Antigen Titres in North American Tertiary Referral Liver Centres." *Liver Int* 33, no. 9 (2013): 1363-9.

40. Ducancelle, A., Abgueguen, P., Birguel, J., Mansour, W., Pivert, A., Le Guillou-Guillemette, H., Sobnangou, J.-J., Rameau, A., Huraux, J.-M., and Lunel-Fabiani, F. "High Endemicity and Low Molecular Diversity of Hepatitis B Virus Infections in Pregnant Women in a Rural District of North Cameroon." *PLoS One* 8, no. 11 (2013): e80346.

41. Komas, N. P., Vickos, U., Hübschen, J. M., Béré, A., Manirakiza, A., Muller, C. P., and Le Faou, A. "Cross-Sectional Study of Hepatitis B Virus Infection in Rural Communities, Central African Republic." *BMC Infect Dis* 13, no. 1 (2013): 286.

42. Di Lello, F. A., Pineiro, Y. L. F. G., Munoz, G., and Campos, R. H. "Diversity of Hepatitis B and C Viruses in Chile." *J Med Virol* 81, no. 11 (2009): 1887-94.

43. Venegas, M., Alvarado-Mora, M. V., Villanueva, R. A., Rebello Pinho, J. R., Carrilho, F. J., Locarnini, S., Yuen, L., and Brahm, J. "Phylogenetic Analysis of Hepatitis B Virus Genotype F Complete Genome Sequences from Chilean Patients with Chronic Infection." *J Med Virol* 83, no. 9 (2011): 1530-6.

44. Li, H.-M., Wang, J.-Q., Wang, R., Zhao, Q., Li, L., Zhang, J.-P., and Shen, T. "Hepatitis B Virus Genotypes and Genome Characteristics in China." *World journal of gastroenterology: WJG* 21, no. 21 (2015): 6684.

45. Zhong, Y.-W., Li, J., Song, H.-B., Duan, Z.-P., Dong, Y., Xing, X.-Y., Li, X.-D., Gu, M.-L., Han, Y.-K., and Zhu, S.-S. "Virologic and Clinical Characteristics of Hbv Genotypes/Subgenotypes in 487 Chinese Pediatric Patients with Chb." *BMC Infect Dis* 11, no. 1 (2011): 262.

46. Chu, C. J., Hussain, M., and Lok, A. S. F. "Hepatitis B Virus Genotype B Is Associated with Earlier Hbeag Seroconversion Compared with Hepatitis B Virus Genotype C." *Gastroenterology* 122, no. 7 (2002): 1756-62.

47. Nie, J., Li, J., Sun, K., Sun, M., Chen, J., Ma, J., Yan, L., and Zhuang, H. "Hbv/D1: A Major Hbv Subgenotype Circulating in Uyghur Patients with Chronic Hbv Infection in Xinjiang, China." *Arch Virol* 157, no. 8 (2012): 1541-49.

48. Alvarado Mora, M. V., Romano, C. M., Gomes-Gouvêa, M. S., Gutierrez, M. F., Botelho, L., Carrilho, F. J., and Pinho, J. R. R. "Molecular Characterization of the Hepatitis B Virus Genotypes in Colombia: A Bayesian Inference on the Genotype F." *Infection, genetics and Evolution* 11, no. 1 (2011): 103-08.

49. Gulube, Z., Chirara, M., Kew, M., Tanaka, Y., Mizokami, M., and Kramvis, A. "Molecular Characterization of Hepatitis B Virus Isolates from Zimbabwean Blood Donors." *Journal of medical virology* 83, no. 2 (2011): 235-44.

50. Cortes-Mancera, F., Loureiro, C. L., Hoyos, S., Restrepo, J. C., Correa, G., Jaramillo, S., Norder, H., Pujol, F. H., and Navas, M. C. "Etiology and Viral Genotype in Patients with End-Stage Liver Diseases Admitted to a Hepatology Unit in Colombia." *Hepat Res Treat* 2011 (2011): 363205.

51. Devesa, M., Loureiro, C. L., Rivas, Y., Monsalve, F., Cardona, N., Duarte, M. C., Poblete, F., Gutierrez, M. F., Botto, C., and Pujol, F. H. "Subgenotype Diversity of Hepatitis B Virus American Genotype F in Amerindians from Venezuela and the General Population of Colombia." *J Med Virol* 80, no. 1 (2008): 20-6.

52. Angounda, B. M., Ngouloubi, G. H., Dzia, A. B., Boumba, L. M. A., Baha, W., Moukassa, D., Ahombo, G., Ennaji, M. M., and Ibara, J. R. "Molecular Characterization of Hepatitis B Virus among Chronic Hepatitis B Patients from Pointe Noire, Republic of Congo." *Infect Agent Cancer* 11 (2016): 51.

53. Leon, B., Taylor, L., Vargas, M., Luftig, R. B., Albertazzi, F., Herrero, L., and Visona, K. "Hbx M130k and V131i (T-a) Mutations in Hbv Genotype F During a Follow-up Study in Chronic Carriers." *Virol J* 2 (2005): 60.

54. Deterding, K., Constantinescu, I., Nedelcu, F. D., Gervain, J., Nemecek, V., Srtunecky, O., Vince, A., Grgurevic, I., Bielawski, K. P., Zalewska, M., Bock, T., Ambrozaitis, A., Stanczak, J., Takacs, M., Chulanov, V., Slusarczyk, J., Drazd'akova, M., Wiegand, J., Cornberg, M., Manns, M. P., and Wedemeyer, H. "Prevalence of Hbv Genotypes in Central and Eastern Europe." *J Med Virol* 80, no. 10 (2008): 1707-11.

55. Loureiro, C. L., Aguilar, J. C., Aguiar, J., Muzio, V., Pentón, E., Garcia, D., Guillen, G., and Pujol, F. H. "Hbv Genotypic Variability in Cuba." *PLoS One* 10, no. 3 (2015): e0118959.

56. Arikan, A., Şanlidağ, T., Süer, K., Sayan, M., Akçali, S., and Güler, E. "Molecular Epidemiology of Hepatitis B Virus in Northern Cyprus." *Mikrobiyol Bul* 50, no. 1 (2016): 86-93.

57. Shindano, T. A., Horsmans, Y., and Kabamba, B. M. "Genetic and Phylogenic Characterization of Hepatitis B Virus in the Eastern Part of the Democratic Republic of Congo." *J Med Virol* 90, no. 2 (2018): 250-54.

58. Krarup, H., Andersen, S., Madsen, P. H., Christensen, P. B., Laursen, A. L., Bentzen-Petersen, A., Møller, J. M., Weis, N., and Group, D. "Hbeag and Not Genotypes Predicts Viral Load in Patients with Hepatitis B in Denmark: A Nationwide Cohort Study." *Scand J Gastroenterol* 46, no. 12 (2011): 1484-91.

59. Khaled, I. A., Mahmoud, O. M., Saleh, A. F., and Bioumie, E. E. "Prevalence of Hbv Genotypes among Egyptian Hepatitis Patients." *Mol Biol Rep* 38, no. 7 (2011): 4353-7.

60. Saudy, N., Sugauchi, F., Tanaka, Y., Suzuki, S., Aal, A. A., Zaid, M. A., Agha, S., and Mizokami, M. "Genotypes and Phylogenetic Characterization of Hepatitis B and Delta Viruses in Egypt." *J Med Virol* 70, no. 4 (2003): 529-36.

61. Habil, F. E., Mahdi, W. K., Abdelwahab, S. F., and Abdel-Hamid, M. "Hepatitis B Virus Genotype D Predominates Hbsag-Positive Egyptian Blood Donors and Is Mainly Associated with a Negative Hbeag Serostatus." *Intervirology* 56, no. 5 (2013): 278-83.

62. Palumbo, E., Scotto, G., Faleo, G., Cibelli, D. C., Saracino, A., and Angarano, G. "Prevalence of Hbv-Genotypes in Immigrants Affected by Hbv-Related Chronic Active Hepatitis." *Arq Gastroenterol* 44, no. 1 (2007): 54-57.

63. Hundie, G., Stalin Raj, V., Gebre Michael, D., Pas, S., Koopmans, M., Osterhaus, A., Smits, S., and Haagmans, B. "A Novel Hepatitis B Virus Subgenotype D10 Circulating in Ethiopia." *J Viral Hepat* 24, no. 2 (2017): 163-73.

64. Jazayeri, M. S., Basuni, A. A., Cooksley, G., Locarnini, S., and Carman, W. F. "Hepatitis B Virus Genotypes, Core Gene Variability and Ethnicity in the Pacific Region." *J Hepatol* 41, no. 1 (2004): 139-46.

65. Stuyver, L., De Gendt, S., Van Geyt, C., Zoulim, F., Fried, M., Schinazi, R. F., and Rossau, R. "A New Genotype of Hepatitis B Virus: Complete Genome and Phylogenetic Relatedness." *J Gen Virol* 81, no. Pt 1 (2000): 67-74.

66. Trimoulet, P., Boutonnet, M., Winnock, M., Faure, M., Loko, M.-A., De Lédinghen, V., Bernard, P.-H., Castéra, L., Foucher, J., Dupon, M., Ragnaud, J.-M., Lafon, M.-É., Couzigou, P., Dabis, F., Fleury, H., and Neau, D. "Hepatitis B Virus Genotypes : A Retrospective Survey in Southwestern France, 1999-2004." *Gastroenterol Clin Biol* 31, no. 12 (2007): 1088-94.

67. Halfon, P., Bourliere, M., Pol, S., Benhamou, Y., Ouzan, D., Rotily, M., Khiri, H., Renou, C., Penaranda, G., and Saadoun, D. "Multicentre Study of Hepatitis B Virus Genotypes in France: Correlation with Liver Fibrosis and Hepatitis B E Antigen Status." *J Viral Hepat* 13, no. 5 (2006): 329-35.

68. Ganne‐Carrié, N., Williams, V., Kaddouri, H., Trinchet, J. C., Dziri‐Mendil, S., Alloui, C., Hawajri, N. A., Dény, P., Beaugrand, M., and Gordien, E. "Significance of Hepatitis B Virus Genotypes a to E in a Cohort of Patients with Chronic Hepatitis B in the Seine Saint Denis District of Paris (France)." *Journal of medical virology* 78, no. 3 (2006): 335-40.

69. Makuwa, M., Caron, M., Souquiere, S., Malonga-Mouelet, G., Mahé, A., and Kazanji, M. "Prevalence and Genetic Diversity of Hepatitis B and Delta Viruses in Pregnant Women in Gabon: Molecular Evidence That Hepatitis Delta Virus Clade 8 Originates from and Is Endemic in Central Africa." *J Clin Microbiol* 46, no. 2 (2008): 754-56.

70. Shimakawa, Y., Lemoine, M., Njai, H. F., Bottomley, C., Ndow, G., Goldin, R. D., Jatta, A., Jeng-Barry, A., Wegmuller, R., and Moore, S. E. "Natural History of Chronic Hbv Infection in West Africa: A Longitudinal Population-Based Study from the Gambia." *Gut* (2015): gutjnl-2015-309892.

71. Bonney, J. H. K., Osei-Kwasi, M., Adiku, T. K., Barnor, J. S., Amesiya, R., Kubio, C., Ahadzie, L., Ölschläger, S., Lelke, M., and Becker-Ziaja, B. "Hospital-Based Surveillance for Viral Hemorrhagic Fevers and Hepatitides in Ghana." *PLoS Negl Trop Dis* 7, no. 9 (2013): e2435.

72. Huy, T. T., Ishikawa, K., Ampofo, W., Izumi, T., Nakajima, A., Ansah, J., Tetteh, J. O., Nii-Trebi, N., Aidoo, S., Ofori-Adjei, D., Sata, T., Ushijima, H., and Abe, K. "Characteristics of Hepatitis B Virus in Ghana: Full Length Genome Sequences Indicate the Endemicity of Genotype E in West Africa." *J Med Virol* 78, no. 2 (2006): 178-84.

73. Fylaktou, A., Papaventsis, D., Daoudaki, M., Moskophidis, M., Reiberger, T., and Malisiovas, N. "Molecular Epidemiology of Chronic Hepatitis B Virus Infection in Greece." *J Med Virol* 83, no. 2 (2011): 245-52.

74. Garmiri, P., Loua, A., Haba, N., Candotti, D., and Allain, J.-P. "Deletions and Recombinations in the Core Region of Hepatitis B Virus Genotype E Strains from Asymptomatic Blood Donors in Guinea, West Africa." *Journal of General Virology* 90, no. 10 (2009): 2442-51.

75. Andernach, I. E., Nolte, C., Pape, J. W., and Muller, C. P. "Slave Trade and Hepatitis B Virus Genotypes and Subgenotypes in Haiti And africa." *Emerging Infectious Diseases* 15, no. 8 (2009): 1222-28.

76. Björnsdottir, T. B., Stanzeit, B., Sällberg, M., Löve, A., and Hultgren, C. "Changing Prevalence of Hepatitis B Virus Genotypes in Iceland." *Journal of medical virology* 77, no. 4 (2005): 481-85.

77. Ismail, A. M., Puhazhenthi, K. S., Sivakumar, J., Eapen, C. E., Kannangai, R., and Abraham, P. "Molecular Epidemiology and Genetic Characterization of Hepatitis B Virus in the Indian Subcontinent." *Int J Infect Dis* 20 (2014): 1-10.

78. Gandhe, S. S., Chadha, M. S., and Arankalle, V. A. "Hepatitis B Virus Genotypes and Serotypes in Western India: Lack of Clinical Significance." *Journal of medical virology* 69, no. 3 (2003): 324-30.

79. Vivekanandan, P., Abraham, P., Sridharan, G., Chandy, G., Daniel, D., Raghuraman, S., Daniel, H. D., and Subramaniam, T. "Distribution of Hepatitis B Virus Genotypes in Blood Donors and Chronically Infected Patients in a Tertiary Care Hospital in Southern India." *Clinical Infectious Diseases* 38, no. 9 (2004): e81-e86.

80. Pal, A., Panigrahi, R., Biswas, A., Datta, S., Sarkar, N., Guha, S. K., Saha, B., Banerjee, A., Chakrabarti, S., and Chakravarty, R. "Influence of Hiv-Associated Degree of Immune Suppression on Molecular Heterogeneity of Hepatitis B Virus among Hiv Co-Infected Patients." *Virology* 436, no. 1 (2013): 134-42.

81. Ghosh, S., Banerjee, P., Deny, P., Mondal, R., Nandi, M., Roychoudhury, A., Das, K., Banerjee, S., Santra, A., and Zoulim, F. "New Hbv Subgenotype D9, a Novel D/C Recombinant, Identified in Patients with Chronic Hbeag‐Negative Infection in Eastern India." *J Viral Hepat* 20, no. 3 (2013): 209-18.

82. Banerjee, P., Mondal, R. K., Nandi, M., Ghosh, S., Khatun, M., Chakraborty, N., Bhattacharya, S., Roychoudhury, A., Banerjee, S., Santra, A., Sil, S., Chowdhury, A., Bhaumik, P., and Datta, S. "A Rare Hbv Subgenotype D4 with Unique Genomic Signatures Identified in North-Eastern India--an Emerging Clinical Challenge?" *PLoS One* 9, no. 10 (2014): e109425.

83. Nurainy, N., Muljono, D. H., Sudoyo, H., and Marzuki, S. "Genetic Study of Hepatitis B Virus in Indonesia Reveals a New Subgenotype of Genotype B in East Nusa Tenggara." *Arch Virol* 153, no. 6 (2008): 1057-65.

84. Prasetyo, A. A., Dirgahayu, P., Sari, Y., Hudiyono, H., and Kageyama, S. "Molecular Epidemiology of Hiv, Hbv, Hcv, and Htlv-1/2 in Drug Abuser Inmates in Central Javan Prisons, Indonesia." *The Journal of Infection in Developing Countries* 7, no. 06 (2013): 453-67.

85. Lusida, M. I., Surayah, Sakugawa, H., Nagano-Fujii, M., Soetjipto, Mulyanto, Handajani, R., Boediwarsono, Setiawan, P. B., Nidom, C. A., Ohgimoto, S., and Hotta, H. "Genotype and Subtype Analyses of Hepatitis B Virus (Hbv) and Possible Co-Infection of Hbv and Hepatitis C Virus (Hcv) or Hepatitis D Virus (Hdv) in Blood Donors, Patients with Chronic Liver Disease and Patients on Hemodialysis in Surabaya, Indonesia." *Microbiol Immunol* 47, no. 12 (2003): 969-75.

86. Jinata, C., Giri-Rachman, E. A., and Retnoningrum, D. S. "Molecular Analysis of Immune-Escape Mutants of Hepatitis B Virus from Local Clinical Samples." *Microbiology Indonesia* 6, no. 1 (2012): 2.

87. Lusida, M. I., Nugrahaputra, V. E., Soetjipto, Handajani, R., Nagano-Fujii, M., Sasayama, M., Utsumi, T., and Hotta, H. "Novel Subgenotypes of Hepatitis B Virus Genotypes C and D in Papua, Indonesia." *J Clin Microbiol* 46, no. 7 (2008): 2160-6.

88. Asli, M., Kandelouei, T., Rahimyan, K., Davoodbeglou, F., and Vaezjalali, M. "Characterization of Occult Hepatitis B Infection among Injecting Drug Users in Tehran, Iran." *Hepat Mon* 16, no. 3 (2016): e34763.

89. Karimi, A., Moezzi, M., and Imani, R. "Sequence-Based Genotyping of Hepatitis B Virus in General Population." *Med J Islam Repub Iran* 29 (2015): 165.

90. Mohammadnejad, L., Farajnia, S., Parivar, K., Naghili, B., and Kheirnagsh, R. Y. "Hepatitis B Virus Genotypes in Eastern Azerbaijan, Northwest Iran." *Arch Iran Med* 15, no. 7 (2012): 446.

91. Karimabad, M. N., Hassanshahi, G., Gharebaghiyan, A., and Sharifi, Z. "Study of Hepatitis B Prevalence in Parallel with the Most Frequent Hbvgenotype in South Iranian Blood Donors." *J Clin Lab Anal* 26, no. 6 (2012): 407-11.

92. Yoosefi, K., Taheri, M., Khosravi, S., Saneie-Moghadam, E., and Mohagheghi-Fard, A. H. "Genotyping of Hepatitis B Virus by Multiplex Pcr in Sistan and Baluchestan Province." *Zahedan Journal of Research in Medical Sciences* In Press, no. InPress (2016).

93. Sharifi, Z., Yari, F., and Gharebaghiyan, A. "Sequence Analysis of the Polymerase Gene in Hepatitis B Virus Infected Blood Donors in Iran." *Arch Iran Med* 15, no. 2 (2012): 88-90.

94. Pourkarim, M. R., Vergote, V., Amini‐Bavil‐Olyaee, S., Sharifi, Z., Sijmons, S., Lemey, P., Maes, P., Alavian, S. M., and Van Ranst, M. "Molecular Characterization of Hepatitis B Virus (Hbv) Strains Circulating in the Northern Coast of the Persian Gulf and Its Comparison with Worldwide Distribution of Hbv Subgenotype D1." *Journal of medical virology* 86, no. 5 (2014): 745-57.

95. Goreal, A. A., and Abdulla, I. M. "Detection of Hepatitis -B Virus Genotypes among Chronic Carriers in Duhok." *J Fac Med Baghdad* 58, no. 2 (2016).

96. Jackson, V., Ferguson, W., Kelleher, T. B., Lawless, M., Eogan, M., Nusgen, U., Coughlan, S., Connell, J., and Lambert, J. S. "Lamivudine Treatment and Outcome in Pregnant Women with High Hepatitis B Viral Loads." *Eur J Clin Microbiol Infect Dis* 34, no. 3 (2015): 619-23.

97. Shirazi, R., Ram, D., Rakovsky, A., Gozlan, Y., Bucris, E., Shaked-Mishan, P., Picard, O., Shemer-Avni, Y., Ben-Zvi, H., and Halutz, O. "Sat-154-Characterization of Hepatitis Delta Infection in Israel: Prevalence and Genotypes." *J Hepatol* 66, no. 1 (2017): S688.

98. Velati, C., Romanò, L., Fomiatti, L., Baruffi, L., and Zanetti, A. R. "Impact of Nucleic Acid Testing for Hepatitis B Virus, Hepatitis C Virus, and Human Immunodeficiency Virus on the Safety of Blood Supply in Italy: A 6‐Year Survey." *Transfusion* 48, no. 10 (2008): 2205-13.

99. Zuccaro, O., Romanò, L., Mele, A., Mariano, A., Clementi, M., Tosti, M. E., Taliani, G., Galli, C., Zanetti, A. R., and Spada, E. "Clinical, Epidemiological and Virological Features of Acute Hepatitis B in Italy." *Infection* 43, no. 4 (2015): 431-41.

100. Medici, M. C., Aloisi, A., Martinelli, M., Abelli, L. A., Casula, F., Valcavi, P., Dettori, G., and Chezzi, C. "Hbv Genotypes and Antiviral-Resistant Variants in Hbv Infected Subjects in Northern Italy." *Microbiologica-Quarterly Journal of Microbiological Sciences* 29, no. 1 (2006): 63-68.

101. Manno, M., Cammà, C., Schepis, F., Bassi, F., Gelmini, R., Giannini, F., Miselli, F., Grottola, A., Ferretti, I., and Vecchi, C. "Natural History of Chronic Hbv Carriers in Northern Italy: Morbidity and Mortality after 30 Years." *Gastroenterology* 127, no. 3 (2004): 756-63.

102. Dal Molin, G., Poli, A., Croce, L. S., D'agaro, P., Biagi, C., Comar, M., Tiribelli, C., and Campello, C. "Hepatitis B Virus Genotypes, Core Promoter Variants, and Precore Stop Codon Variants in Patients Infected Chronically in North-Eastern Italy." *J Med Virol* 78, no. 6 (2006): 734-40.

103. Piermarini, M., Medori, M. C., Pagnani, A., Proietti, M., and Scaccetti, A. "Genotypes of the Hepatitis B Virus within the Area of Terni, Italy: Our Experience." *Microbiologia Medica* 31, no. 3 (2016).

104. Togo, S., Arai, M., Tawada, A., Chiba, T., Kanda, T., Fujiwara, K., Imazeki, F., and Yokosuka, O. "Clinical Importance of Serum Hepatitis B Surface Antigen Levels in Chronic Hepatitis B." *J Viral Hepat* 18, no. 10 (2011): e508-15.

105. Yotsuyanagi, H., Okuse, C., Yasuda, K., Orito, E., Nishiguchi, S., Toyoda, J., Tomita, E., Hino, K., Okita, K., and Murashima, S. "Distinct Geographic Distributions of Hepatitis B Virus Genotypes in Patients with Acute Infection in Japan." *Journal of medical virology* 77, no. 1 (2005): 39-46.

106. Fujie, H., Moriya, K., Shintani, Y., Yotsuyanagi, H., Iino, S., Kimura, S., and Koike, K. "Hepatitis B Virus Genotypes and Hepatocellular Carcinoma in Japan." *Gastroenterology* 120, no. 6 (2001): 1564-65.

107. Takano, T., Tajiri, H., Hosono, S., Inui, A., Murakami, J., Ushijima, K., Miyoshi, Y., Etani, Y., Abukawa, D., Suzuki, M., and Brooks, S. "Natural History of Chronic Hepatitis B Virus Infection in Children in Japan: A Comparison of Mother-to-Child Transmission with Horizontal Transmission." *J Gastroenterol* 52, no. 9 (2017): 1041-50.

108. Imamura, T., Yokosuka, O., Kurihara, T., Kanda, T., Fukai, K., Imazeki, F., and Saisho, H. "Distribution of Hepatitis B Viral Genotypes and Mutations in the Core Promoter and Precore Regions in Acute Forms of Liver Disease in Patients from Chiba, Japan." *Gut* 52, no. 11 (2003): 1630-37.

109. Matsuura, K., Tanaka, Y., Hige, S., Yamada, G., Murawaki, Y., Komatsu, M., Kuramitsu, T., Kawata, S., Tanaka, E., Izumi, N., Okuse, C., Kakumu, S., Okanoue, T., Hino, K., Hiasa, Y., Sata, M., Maeshiro, T., Sugauchi, F., Nojiri, S., Joh, T., Miyakawa, Y., and Mizokami, M. "Distribution of Hepatitis B Virus Genotypes among Patients with Chronic Infection in Japan Shifting toward an Increase of Genotype A." *J Clin Microbiol* 47, no. 5 (2009): 1476-83.

110. Masaadeh, H. A., Hayajneh, W. A., and Alqudah, E. A. "Hepatitis B Virus Genotypes and Lamivudine Resistance Mutations in Jordan." *World J Gastroenterol* 14, no. 47 (2008): 7231-4.

111. Останкова, Ю., Семенов, А., Буркитбаев, Ж., and Савчук, Т. "Тотолян Арег А. Генетические Варианты Вируса Гепатита B У Первичных Доноров В Г. Астана, Казахстан." *Инфекция и иммунитет* 6, no. 4 (2016): 359-66.

112. Ngoi, C. N., Siqueira, J., Li, L., Deng, X., Mugo, P., Graham, S. M., Price, M. A., Sanders, E. J., and Delwart, E. "The Plasma Virome of Febrile Adult Kenyans Shows Frequent Parvovirus B19 Infections and a Novel Arbovirus (Kadipiro Virus)." *Journal of General Virology* 97, no. 12 (2016): 3359-67.

113. Mabeya, S. N., Ngugi, C., Lihana, R. W., Khamadi, S. A., and Nyamache, A. K. "Predominance of Hepatitis B Virus Genotype a among Treated Hiv Infected Patients Experiencing High Hepatitis B Virus Drug Resistance in Nairobi, Kenya." *AIDS Res Hum Retroviruses* 33, no. 9 (2017): 966-69.

114. Ochwoto, M., Chauhan, R., Gopalakrishnan, D., Chen, C.-Y., Okoth, F., Kioko, H., Kimotho, J., Kaiguri, P., and Kramvis, A. "Genotyping and Molecular Characterization of Hepatitis B Virus in Liver Disease Patients in Kenya." *Infection, genetics and Evolution* 20 (2013): 103-10.

115. Kwange, S. O., Budambula, N. L., Kiptoo, M. K., Okoth, F., Ochwoto, M., Oduor, M., and Kimotho, J. H. "Hepatitis B Virus Subgenotype A1, Occurrence of Subgenotype D4, and S Gene Mutations among Voluntary Blood Donors in Kenya." *Virus Genes* 47, no. 3 (2013): 448-55.

116. Semenov, A. V., Ostankova, Y. V., Nogoybaeva, K. A., Kasymbekova, K. T., Lavrentieva, I. N., Tobokalova, S. T., and Totolian, A. A. "Molecular Epidemiology Features of Hbv/Hdv Co-Infection in Kyrgyzstan." *Russian Journal of Infection and Immunity* 6, no. 2 (2016): 141-50.

117. Olinger, C. M., Jutavijittum, P., Hübschen, J. M., Yousukh, A., Samountry, B., Thammavong, T., Toriyama, K., and Muller, C. P. "Possible New Hepatitis B Virus Genotype, Southeast Asia." *Emerging Infectious Diseases* 14, no. 11 (2008): 1777.

118. El Chaar, M., El Jisr, T., and Allain, J. P. "Hepatitis B Virus DNA Splicing in Lebanese Blood Donors and Genotype a to E Strains: Implications for Hepatitis B Virus DNA Quantification and Infectivity." *J Clin Microbiol* 50, no. 10 (2012): 3159-67.

119. Salem, M. A., Elnifro, E. M., and Alshuwen, F. "Molecular Analysis of Hepatitis B Virus Isolates in Libya: Predominant Circulation of Hepatitis B Virus Genotype D." *Journal of Gastroenterology and Hepatology Research* 1, no. 7 (2012): 119-21.

120. Dupinay, T., Restorp, K., Leutscher, P., Rousset, D., Chemin, I., Migliani, R., Magnius, L., and Norder, H. "High Prevalence of Hepatitis B Virus Genotype E in Northern Madagascar Indicates a West-African Lineage." *J Med Virol* 82, no. 9 (2010): 1515-26.

121. Andriamandimby, S. F., Lo Presti, A., Lai, A., Olive, M. M., Angeletti, S., De Florio, L., Cella, E., Razafindramparany, M., Ravalohery, J. P., Andriamamonjy, S., Gioffre, S., Zehender, G., Mottini, G., Ciccozzi, M., and Heraud, J. M. "Genetic Diversity of Hepatitis B Virus (Hbv) in Madagascar." *J Med Virol* 88, no. 12 (2016): 2138-44.

122. Galluzzo, C., Liotta, G., Andreotti, M., Luhanga, R., Jere, H., Mancinelli, S., Maulidi, M., Sagno, J. B., Pirillo, M., and Erba, F. "Emergence of Lamivudine Resistance Hepatitis B Virus Mutations in Pregnant Women Infected with Hbv and Hiv Receiving Antiretroviral Prophylaxis for the Prevention of Mother‐to‐Infant Transmission in Malawi." *Journal of medical virology* 84, no. 10 (2012): 1553-57.

123. Aoudjane, S., Chaponda, M., González Del Castillo, A. A., O'connor, J., Noguera, M., Beloukas, A., Hopkins, M., Khoo, S., Van Oosterhout, J. J., and Geretti, A. M. "Hepatitis B Virus Sub-Genotype A1 Infection Is Characterized by High Replication Levels and Rapid Emergence of Drug Resistance in Hiv-Positive Adults Receiving First-Line Antiretroviral Therapy in Malawi." *Clinical Infectious Diseases* 59, no. 11 (2014): 1618-26.

124. Meldal, B. H., Bon, A. H., Prati, D., Ayob, Y., and Allain, J. P. "Diversity of Hepatitis B Virus Infecting Malaysian Candidate Blood Donors Is Driven by Viral and Host Factors." *J Viral Hepat* 18, no. 2 (2011): 91-101.

125. Lim, C. K., Tan, J. T. M., Khoo, J. B. S., Ravichandran, A., Low, H. M., Chan, Y. C., and Ton, S. H. "Correlations of Hbv Genotypes, Mutations Affecting Hbeag Expression and Hbeag/ Anti-Hbe Status in Hbv Carriers." *Int J Med Sci* 3, no. 1 (2006): 14-20.

126. Cella, E., Ceccarelli, G., Vita, S., Lai, A., Presti, A. L., Blasi, A., Palco, M. L., Guarino, M. P. L., Zehender, G., and Angeletti, S. "First Epidemiological and Phylogenetic Analysis of Hepatitis B Virus Infection in Migrants from Mali." *Journal of medical virology* 89, no. 4 (2017): 639-46.

127. Mansour, W., Malick, F., Fall, Z., Sidiya, A., Ishagh, E., Chekaraou, M. A., Veillon, P., Ducancelle, A., Brichler, S., and Le Gal, F. "Prevalence, Risk Factors, and Molecular Epidemiology of Hepatitis B and Hepatitis Delta Virus in Pregnant Women and in Patients in Mauritania." *Journal of medical virology* 84, no. 8 (2012): 1186-98.

128. Ruiz-Tachiquin, M. E., Valdez-Salazar, H. A., Juarez-Barreto, V., Dehesa-Violante, M., Torres, J., Munoz-Hernandez, O., and Alvarez-Munoz, M. T. "Molecular Analysis of Hepatitis B Virus "a" Determinant in Asymptomatic and Symptomatic Mexican Carriers." *Virol J* 4 (2007): 6.

129. Mata Marin, J. A., Arroyo Anduiza, C. I., Calderon, G. M., Cazares Rodriguez, S., Fuentes Allen, J. L., Arias Flores, R., and Gaytan Martinez, J. "Prevalence and Resistance Pattern of Genotype G and H in Chronic Hepatitis B and Hiv Co-Infected Patients in Mexico." *Ann Hepatol* 11, no. 1 (2012): 47-51.

130. Roman, S., Tanaka, Y., Khan, A., Kurbanov, F., Kato, H., Mizokami, M., and Panduro, A. "Occult Hepatitis B in the Genotype H-Infected Nahuas and Huichol Native Mexican Population." *Journal of medical virology* 82, no. 9 (2010): 1527-36.

131. Sanchez, L. V., Maldonado, M., Bastidas-Ramirez, B. E., Norder, H., and Panduro, A. "Genotypes and S-Gene Variability of Mexican Hepatitis B Virus Strains." *J Med Virol* 68, no. 1 (2002): 24-32.

132. Sanchez, L. V., Tanaka, Y., Maldonado, M., Mizokami, M., and Panduro, A. "Difference of Hepatitis B Virus Genotype Distribution in Two Groups of Mexican Patients with Different Risk Factors. High Prevalence of Genotype H and G." *Intervirology* 50, no. 1 (2007): 9-15.

133. Tsatsralt‐Od, B., Takahashi, M., Endo, K., Agiimaa, D., Buyankhuu, O., Ninomiya, M., Lorenzo, F. R., and Okamoto, H. "Prevalence of Hepatitis B, C, and Delta Virus Infections among Children in Mongolia: Progress in Childhood Immunization." *Journal of medical virology* 79, no. 8 (2007): 1064-74.

134. Takahashi, M., Nishizawa, T., Gotanda, Y., Tsuda, F., Komatsu, F., Kawabata, T., Hasegawa, K., Altankhuu, M., Chimedregzen, U., Narantuya, L., Hoshino, H., Hino, K., Kagawa, Y., and Okamoto, H. "High Prevalence of Antibodies to Hepatitis a and E Viruses and Viremia of Hepatitis B, C, and D Viruses among Apparently Healthy Populations in Mongolia." *Clinical and Vaccine Immunology* 11, no. 2 (2004): 392-98.

135. Tsatsralt-Od, B., Takahashi, M., Nishizawa, T., Endo, K., Inoue, J., and Okamoto, H. "High Prevalence of Dual or Triple Infection of Hepatitis B, C, and Delta Viruses among Patients with Chronic Liver Disease in Mongolia." *J Med Virol* 77, no. 4 (2005): 491-9.

136. Kitab, B., El Feydi, A. E., Afifi, R., Derdabi, O., Cherradi, Y., Benazzouz, M., Rebbani, K., Brahim, I., Alj, H. S., and Zoulim, F. "Hepatitis B Genotypes/Subgenotypes and Mhr Variants among Moroccan Chronic Carriers." *Journal of Infection* 63, no. 1 (2011): 66-75.

137. Baha, W., Ennaji, M. M., Lazar, F., Melloul, M., El Fahime, E., El Malki, A., and Bennani, A. "Hbv Genotypes Prevalence, Precore and Basal Core Mutants in Morocco." *Infection, genetics and Evolution* 12, no. 6 (2012): 1157-62.

138. Cunha, L., Plouzeau, C., Ingrand, P., Gudo, J. P. S., Ingrand, I., Mondlane, J., Beauchant, M., and Agius, G. "Use of Replacement Blood Donors to Study the Epidemiology of Major Blood‐Borne Viruses in the General Population of Maputo, Mozambique." *Journal of medical virology* 79, no. 12 (2007): 1832-40.

139. Nakai, K., Win, K. M., Oo, S. S., Arakawa, Y., and Abe, K. "Molecular Characteristic-Based Epidemiology of Hepatitis B, C, and E Viruses and Gb Virus C/Hepatitis G Virus in Myanmar." *J Clin Microbiol* 39, no. 4 (2001): 1536-9.

140. Tamandjou, C., Kaindjee-Tjituka, F., Brandt, L., Cotton, M., Nel, E., Preiser, W., and Andersson, M. "A Cross-Sectional Study of Hepatitis B Virus Infection in Hiv-Infected Children in Windhoek, Namibia." *BMJ Global Health* 2, no. Suppl 2 (2017): A47-A48.

141. Kramvis, A., Restorp, K., Norder, H., Botha, J. F., Magnius, L. O., and Kew, M. C. "Full Genome Analysis of Hepatitis B Virus Genotype E Strains from South-Western Africa and Madagascar Reveals Low Genetic Variability." *J Med Virol* 77, no. 1 (2005): 47-52.

142. Shrestha, S. M., Shrestha, S., Shrestha, A., Tsuda, F., Endo, K., Takahashi, M., and Okamoto, H. "High Prevalence of Hepatitis B Virus Infection and Inferior Vena Cava Obstruction among Patients with Liver Cirrhosis or Hepatocellular Carcinoma in Nepal." *J Gastroenterol Hepatol* 22, no. 11 (2007): 1921-8.

143. Zoutendijk, R., Zaaijer, H. L., De Vries-Sluijs, T. E., Reijnders, J. G., Mulder, J. W., Kroon, F. P., Richter, C., Van Der Eijk, A. A., Sonneveld, M. J., and Hansen, B. E. "Hepatitis B Surface Antigen Declines and Clearance During Long-Term Tenofovir Therapy in Patients Coinfected with Hbv and Hiv." *J Infect Dis* 206, no. 6 (2012): 974-80.

144. Van Houdt, R., Bruisten, S. M., Koedijk, F. D., Dukers, N. H., Op De Coul, E. L., Mostert, M. C., Niesters, H. G., Richardus, J. H., De Man, R. A., Van Doornum, G. J., Van Den Hoek, J. A., Coutinho, R. A., Van De Laar, M. J., and Boot, H. J. "Molecular Epidemiology of Acute Hepatitis B in the Netherlands in 2004: Nationwide Survey." *J Med Virol* 79, no. 7 (2007): 895-901.

145. Toy, M., Veldhuijzen, I. K., Mostert, M. C., De Man, R. A., and Richardus, J. H. "Transmission Routes of Hepatitis B Virus Infection in Chronic Hepatitis B Patients in the Netherlands." *J Med Virol* 80, no. 3 (2008): 399-404.

146. Mohamed, R., Desmond, P., Suh, D. J., Amarapurkar, D., Gane, E., Guangbi, Y., Hou, J. L., Jafri, W., Lai, C. L., Lee, C. H., Lee, S. D., Lim, S. G., Guan, R., Phiet, P. H., Piratvisuth, T., Sollano, J., and Wu, J. C. "Practical Difficulties in the Management of Hepatitis B in the Asia-Pacific Region." *J Gastroenterol Hepatol* 19, no. 9 (2004): 958-69.

147. Brah, S., Moussa, S., Inoua, A., Alhousseini, D. M., Daou, M., Madougou, B., Romera, M.-H., Hamadou, A., Adehossi, E., and Parola, P. "Molecular Characterization of Hepatitis B Virus from Chronically-Infected Patients in Niamey, Niger." *International Journal of Infectious Diseases* 45 (2016): 18-23.

148. Abdou Chekaraou, M., Brichler, S., Mansour, W., Le Gal, F., Garba, A., Deny, P., and Gordien, E. "A Novel Hepatitis B Virus (Hbv) Subgenotype D (D8) Strain, Resulting from Recombination between Genotypes D and E, Is Circulating in Niger Along with Hbv/E Strains." *J Gen Virol* 91, no. Pt 6 (2010): 1609-20.

149. J. M. Hübschen, P. O. M., J. C. Forbi, J. A. Otegbayo, C. M. Olinger, E. Charpentier and C. P. Muller. "Detection of a New Subgenotype of Hepatitis B Virus Genotype a Incameroon but Not in Neighbouring Nigeria." *European Society of Clinical Microbiology and Infectious Diseases* Journal Compilation (2010): CMI, 17, 88–94.

150. Grant, J., Agbaji, O., Kramvis, A., Yousif, M., Auwal, M., Penugonda, S., Ugoagwu, P., Murphy, R., and Hawkins, C. "Hepatitis B Virus Sequencing and Liver Fibrosis Evaluation in Hiv/Hbv Co-Infected Nigerians." *Trop Med Int Health* 22, no. 6 (2017): 744-54.

151. Al Baqlani, S. A., Sy, B. T., Ratsch, B. A., Al Naamani, K., Al Awaidy, S., Al Busaidy, S., Pauli, G., and Bock, C.-T. "Molecular Epidemiology and Genotyping of Hepatitis B Virus of Hbsag-Positive Patients in Oman." *PLoS One* 9, no. 5 (2014): e97759.

152. Alam, M. M., Zaidi, S. Z., Malik, S. A., Shaukat, S., Naeem, A., Sharif, S., Angez, M., and Butt, J. A. "Molecular Epidemiology of Hepatitis B Virus Genotypes in Pakistan." *BMC Infect Dis* 7 (2007): 115.

153. Hanif, M., Zaidi, P., Habib, S., Ahmed, A., Raza, A., Ahmed, S., Murtaza, S., and Irfan, J. "Study of Genotypes and Subgenotypes of Hepatitis B Virus Prevalent in Big Cities of Pakistan." *African Journal of Microbiology Research* 7, no. 3 (2012): 152-57.

154. Alam, M. M., Zaidi, S. Z., Shaukat, S., Sharif, S., Angez, M., Naeem, A., Saleha, S., Butt, J. A., and Malik, S. A. "Common Genotypes of Hepatitis B Virus Prevalent in Injecting Drug Abusers (Addicts) of North West Frontier Province of Pakistan." *Virol J* 4 (2007): 63.

155. Abdelnabi, Z., Saleh, N., Baraghithi, S., Glebe, D., and Azzeh, M. "Subgenotypes and Mutations in the S and Polymerase Genes of Hepatitis B Virus Carriers in the West Bank, Palestine." *PLoS One* 9, no. 12 (2014): e113821.

156. Martinez, A. A., Zaldivar, Y. Y., Group, C.-N., De Castillo, Z., Ortiz, A. Y., Mendoza, Y., Cristina, J., and Pascale, J. M. "High Diversity of Hepatitis B Virus Genotypes in Panamanian Blood Donors: A Molecular Analysis of New Variants." *PLoS One* 9, no. 8 (2014): e103545.

157. Von Meltzer, M., Vasquez, S., Sun, J., Wendt, U. C., May, A., Gerlich, W. H., Radtke, M., and Schaefer, S. "A New Clade of Hepatitis B Virus Subgenotype F1 from Peru with Unusual Properties." *Virus Genes* 37, no. 2 (2008): 225-30.

158. Sakamoto, T., Tanaka, Y., Orito, E., Clavio, J., Sugauchi, F., Ito, K., Ozasa, A., Quino, A., Ueda, R., and Sollano, J. "Novel Subtypes (Subgenotypes) of Hepatitis B Virus Genotypes B and C among Chronic Liver Disease Patients in the Philippines." *Journal of General Virology* 87, no. 7 (2006): 1873-82.

159. Batoctoy, K. S., Tseng, T. C., Kao, J. H., Quiza, F. E., Garcia, L. H., Sr., and Lao-Tan, J. "Hbv/a and Hbv/C Genotype Predominance among Patients with Chronic Hepatitis B Virus Infection in Cebu City, Philippines." *Hepatol Int* 5, no. 3 (2011): 774-81.

160. Mota, A., Areias, J., and Cardoso, M. F. "Chronic Liver Disease and Cirrhosis among Patients with Hepatitis B Virus Infection in Northern Portugal with Reference to the Viral Genotypes." *J Med Virol* 83, no. 1 (2011): 71-7.

161. Kim, H., Jee, Y. M., Song, B. C., Shin, J. W., Yang, S. H., Mun, H. S., Kim, H. J., Oh, E. J., Yoon, J. H., Kim, Y. J., Lee, H. S., Hwang, E. S., Cha, C. Y., Kook, Y. H., and Kim, B. J. "Molecular Epidemiology of Hepatitis B Virus (Hbv) Genotypes and Serotypes in Patients with Chronic Hbv Infection in Korea." *Intervirology* 50, no. 1 (2007): 52-7.

162. Ahn, S. H., Yuen, L., Han, K. H., Littlejohn, M., Chang, H. Y., Damerow, H., Ayres, A., Heo, J., Locarnini, S., and Revill, P. A. "Molecular and Clinical Characteristics of Hepatitis B Virus in Korea." *J Med Virol* 82, no. 7 (2010): 1126-34.

163. Gori, A., Gheorgita, S., Spinu, C., Pinzaru, I., Halacu, A., Sajen, O., Suveica, L., Sausy, A., Muller, C. P., and Hubschen, J. M. "Hepatitis B, C and D Virus Genotypes Detected in Hbsag- or Anti-Hcv-Positive People from the Republic of Moldova." *Arch Virol* 163, no. 2 (2018): 431-38.

164. Constantinescu, I., Dinu, A. A., Boscaiu, V., and Niculescu, M. "Hepatitis B Virus Core Promoter Mutations in Patients with Chronic Hepatitis B and Hepatocellular Carcinoma in Bucharest, Romania." *Hepat Mon* 14, no. 10 (2014): e22072.

165. Caruntu, F. A. "M1904 Prevalence of Hbeag and Hbv Genotypes in Romania." *Gastroenterology* 134, no. 4 (2008).

166. Flodgren, E., Bengtsson, S., Knutsson, M., Strebkova, E. A., Kidd, A. H., Alexeyev, O. A., and Kidd-Ljunggren, K. "Recent High Incidence of Fulminant Hepatitis in Samara, Russia: Molecular Analysis of Prevailing Hepatitis B and D Virus Strains." *J Clin Microbiol* 38, no. 9 (2000): 3311-6.

167. Nikitina, G. Y., Semenenko, T. A., Gotvyanskaya, T. P., I.B., H., M.V., K., O.G., N., L.V., Y., L.K., K., and A.P., S. "The Prevalence of Parenteral Hepatitis Markers among the Medical Personnel in the Russian Federation Regions with Different Intensity of Epidemic Process." *KMAX* 19 (2017): 161-67.

168. Klushkina, V. V., Kyuregyan, K. K., Kozhanova, T. V., Popova, O. E., Dubrovina, P. G., Isaeva, O. V., Gordeychuk, I. V., and Mikhailov, M. I. "Impact of Universal Hepatitis B Vaccination on Prevalence, Infection-Associated Morbidity and Mortality, and Circulation of Immune Escape Variants in Russia." *PLoS One* 11, no. 6 (2016): e0157161.

169. Hübschen, J. M., Mugabo, J., Peltier, C. A., Karasi, J. C., Sausy, A., Kirpach, P., Arendt, V., and Muller, C. P. "Exceptional Genetic Variability of Hepatitis B Virus Indicates That Rwanda Is East of an Emerging African Genotype E/A1 Divide." *Journal of medical virology* 81, no. 3 (2009): 435-40.

170. Abdo, A. A., Al-Jarallah, B. M., Sanai, F. M., Hersi, A. S., Al-Swat, K., Azzam, N. A., Al-Dukhayil, M., Al-Maarik, A., and Al-Faleh, F. Z. "Hepatitis B Genotypes: Relation to Clinical Outcome in Patients with Chronic Hepatitis B in Saudi Arabia." *World J Gastroenterol* 12, no. 43 (2006): 7019-24.

171. Vray, M., Debonne, J. M., Sire, J. M., Tran, N., Chevalier, B., Plantier, J. C., Fall, F., Vernet, G., Simon, F., and Mb, P. S. "Molecular Epidemiology of Hepatitis B Virus in Dakar, Senegal." *Journal of medical virology* 78, no. 3 (2006): 329-34.

172. Lazarevic, I., Cupic, M., Delic, D., Svirtlih, N. S., Simonovic, J., and Jovanovic, T. "Distribution of Hbv Genotypes, Subgenotypes and Hbsag Subtypes among Chronically Infected Patients in Serbia." *Arch Virol* 152, no. 11 (2007): 2017-25.

173. Lunacek, N. K., Poljak, M., Meglic-Volkar, J., Rajter, M., Prah, J., Lesnicar, G., Kurincic, T. S., Baklan, Z., Remec, T., and Pal, E. "Epidemiological, Virological and Clinical Characteristics of Hepatitis B Virus Genotypes in Chronically Infected Persons in Slovenia." *Hepatitis Monthly* 17, no. 3 (2017).

174. Utsumi, T., Yano, Y., Truong, B. X., Tanaka, Y., Mizokami, M., Seo, Y., Kasuga, M., Kawabata, M., and Hayashi, Y. "Molecular Epidemiological Study of Hepatitis B Virus Infection in Two Different Ethnic Populations from the Solomon Islands." *J Med Virol* 79, no. 3 (2007): 229-35.

175. Makondo, E., Bell, T. G., and Kramvis, A. "Genotyping and Molecular Characterization of Hepatitis B Virus from Human Immunodeficiency Virus-Infected Individuals in Southern Africa." *PLoS One* 7, no. 9 (2012): e46345.

176. Andersson, M., Maponga, T., Ijaz, S., Barnes, J., Theron, G., Meredith, S., Preiser, W., and Tedder, R. "The Epidemiology of Hepatitis B Virus Infection in Hiv-Infected and Hiv-Uninfected Pregnant Women in the Western Cape, South Africa." *Vaccine* 31, no. 47 (2013): 5579-84.

177. Mahgoub, S., Candotti, D., El Ekiaby, M., and Allain, J. P. "Hepatitis B Virus (Hbv) Infection and Recombination between Hbv Genotypes D and E in Asymptomatic Blood Donors from Khartoum, Sudan." *J Clin Microbiol* 49, no. 1 (2011): 298-306.

178. Echevarria, J. M., and Leon, P. "Hepatitis B Virus Genotypes Identified by a Line Probe Assay (Lipa) among Chronic Carriers from Spain." *Enferm Infecc Microbiol Clin* 22, no. 8 (2004): 452-4.

179. Sanchez-Tapias, J. M., Costa, J., Mas, A., Bruguera, M., and Rodes, J. "Influence of Hepatitis B Virus Genotype on the Long-Term Outcome of Chronic Hepatitis B in Western Patients." *Gastroenterology* 123, no. 6 (2002): 1848-56.

180. Gonzalez, R., Torres, P., Castro, E., Barbolla, L., Candotti, D., Koppelman, M., Zaaijer, H. L., Lelie, N., Allain, J. P., and Echevarria, J. M. "Efficacy of Hepatitis B Virus (Hbv) DNA Screening and Characterization of Acute and Occult Hbv Infections among Blood Donors from Madrid, Spain." *Transfusion* 50, no. 1 (2010): 221-30.

181. Poves-Martínez, E., Del Pozo-Prieto, D., Costero-Pastor, B., Borrego-Rodriguez, G., Beceiro-Pedroño, I., Sanz-García, C., Busteros-Buraza, J. I., and González Palacios, M. "Diagnostic Incidence of the Presence of Positive Hbsag: Epidemiologic, Clinical, and Virological Characteristics." *Revista Espanola de Enfermedades Digestivas* 104, no. 1 (2012): 10.

182. Manamperi, A., Gunawardene, N. S., Wellawatta, C., Abeyewickreme, W., and De Silva, H. J. "Hepatitis B Virus (Hbv) Genotypes in a Group of Sri Lankan Patients with Chronic Infection." *Trop Biomed* 28, no. 2 (2011): 320-4.

183. Yousif, M., Mudawi, H., Hussein, W., Mukhtar, M., Nemeri, O., Glebe, D., and Kramvis, A. "Genotyping and Virological Characteristics of Hepatitis B Virus in Hiv-Infected Individuals in Sudan." *International Journal of Infectious Diseases* 29 (2014): 125-32.

184. Yousif, M., Mudawi, H., Bakhiet, S., Glebe, D., and Kramvis, A. "Molecular Characterization of Hepatitis B Virus in Liver Disease Patients and Asymptomatic Carriers of the Virus in Sudan." *BMC Infect Dis* 13, no. 1 (2013): 328.

185. Lindh, M., Horal, P., and Norkrans, G. "Acute Hepatitis B in Western Sweden–Genotypes and Transmission Routes." *Infection* 28, no. 3 (2000): 161-63.

186. Hirzel, C., Wandeler, G., Owczarek, M., Gorgievski-Hrisoho, M., Dufour, J.-F., Semmo, N., and Zürcher, S. "Molecular Epidemiology of Hepatitis B Virus Infection in Switzerland: A Retrospective Cohort Study." *BMC Infect Dis* 15, no. 1 (2015): 483.

187. Antaki, N., Haffar, S., Ali Deeb, S., Assaad, F., Abou Harb, R., Zeibane, N., Nasserelddine, M., Ibrahim, N., Alhaj, N., Jabbour, E., Aaraj, R., Antaki, F., Kebbewar, K., and Syrian Working Group for the Study of Viral, H. "High Prevalence of Hbv Genotype D in Syria and the Clinical Characteristics of Hepatitis B E Antigen-Negative Chronic Hepatitis B." *Epidemiol Infect* 138, no. 1 (2010): 40-4.

188. Khan, A., Kurbanov, F., Tanaka, Y., Elkady, A., Sugiyama, M., Dustov, A., and Mizokami, M. "Epidemiological and Clinical Evaluation of Hepatitis B, Hepatitis C, and Delta Hepatitis Viruses in Tajikistan." *Journal of medical virology* 80, no. 2 (2008): 268-76.

189. Jutavijittum, P., Jiviriyawat, Y., Yousukh, A., Kunachiwa, W., and Toriyama, K. "Genotypes of Hepatitis B Virus among Voluntary Blood Donors in Northern Thailand." *Hepatology research* 35, no. 4 (2006): 263-66.

190. Jutavijittum, P., Yousukh, A., Jiviriyawat, Y., Kunachiwa, W., and Toriyama, K. "Genotypes of Hepatitis B Virus among Children in Chiang Mai, Thailand." *Southeast Asian J Trop Med Public Health* 39, no. 3 (2008): 394-7.

191. Tangkijvanich, P., Mahachai, V., Komolmit, P., Fongsarun, J., Theamboonlers, A., and Poovorawan, Y. "Hepatitis B Virus Genotypes and Hepatocellular Carcinoma in Thailand." *World J Gastroenterol* 11, no. 15 (2005): 2238-43.

192. Patassi, A., Benaboud, S., Landoh, D. E., Salou, M., Dagnra, A. C., Saka, B., Krivine, A., Meritet, J. F., Pitche, P., and Salmon-Ceron, D. "Hepatitis B Infection in Hiv-1-Infected Patients Receiving Highly Active Antiretroviral Therapy in Lome, Togo: Prevalence and Molecular Consequences." *S Afr Med J* 106, no. 6 (2016).

193. Sayan, M., Akhan, S., Sargin, F., Yasar, K., Cagatay, A., Inan, D., Simsek, F., Kaptan, F., Celikbas, A. K., and Demiraslan, H. "Molecular Characterization of Hiv-1 in Hbv±Hdv/Hcv Co-Infected Hiv-1 Positive Patients in Turkey." *Journal of Clinical Virology* 82 (2016): S87-S88.

194. Altindis, M., Aslan, F. G., Köroglu, M., Ayla, E., Demir, L., Uslan, M. I., Aslan, S., Özdemir, M., and Baykan, M. "Hepatitis B Virus Carrying Drug-Resistance Compensatory Mutations in Chronically Infected Treatment-Naive Patients." *Viral Hepatit Dergisis* 22, no. 3 (2016): 103.

195. Sunbul, M., and Leblebicioglu, H. "Distribution of Hepatitis B Virus Genotypes in Patients with Chronic Hepatitis B in Turkey." *World J Gastroenterol* 11, no. 13 (2005): 1976-80.

196. Atalay, M. A., Gokahmetoglu, S., and Aygen, B. "Genotypes of Hepatitis B Virus in Central Anatolia, Kayseri, Turkey." *Saudi Med J* 32, no. 4 (2011): 360-3.

197. Stepchenkova, T., Karnets, I., Ashworth, K., and Cheusova, T. "The Results of a Study on the Prevalence of Hiv, Hcv and Hbv Genotypes in Some Regions of Ukraine." *Retrovirology* 9, no. 1 (2012): P55.

198. Tedder, R. S., Rodger, A. J., Fries, L., Ijaz, S., Thursz, M., Rosenberg, W., Naoumov, N., Banatvala, J., Williams, R., and Dusheiko, G. "The Diversity and Management of Chronic Hepatitis B Virus Infections in the United Kingdom: A Wake-up Call." *Clinical Infectious Diseases* 56, no. 7 (2012): 951-60.

199. Forbi, J. C., Dillon, M., Purdy, M. A., Drammeh, B. S., Tejada-Strop, A., Mcgovern, D., Xia, G. L., Lin, Y., Ganova-Raeva, L. M., Campo, D. S., Thai, H., Vaughan, G., Haule, D., Kutaga, R. P., Basavaraju, S. V., Kamili, S., and Khudyakov, Y. E. "Molecular Epidemiology of Hepatitis B Virus Infection in Tanzania." *J Gen Virol* (2017).

200. Chu, C. J., Keeffe, E. B., Han, S. H., Perrillo, R. P., Min, A. D., Soldevila-Pico, C., Carey, W., Brown, R. S., Luketic, V. A., Terrault, N., and Lok, A. S. F. "Hepatitis B Virus Genotypes in the United States: Results of a Nationwide Study." *Gastroenterology* 125, no. 2 (2003): 444-51.

201. Hussain, M., Chu, C. J., Sablon, E., and Lok, A. S. F. "Rapid and Sensitive Assays for Determination of Hepatitis B Virus (Hbv) Genotypes and Detection of Hbv Precore and Core Promoter Variants." *J Clin Microbiol* 41, no. 8 (2003): 3699-705.

202. Kato, H., Ruzibakiev, R., Yuldasheva, N., Hegay, T., Kurbanov, F., Achundjanov, B., Tuichiev, L., Usuda, S., Ueda, R., and Mizokami, M. "Hepatitis B Virus Genotypes in Uzbekistan and Validity of Two Different Systems for Genotyping." *Journal of medical virology* 67, no. 4 (2002): 477-83.

203. Avazova, D., Kurbanov, F., Tanaka, Y., Sugiyama, M., Radchenko, I., Ruziev, D., Musabaev, E., and Mizokami, M. "Hepatitis B Virus Transmission Pattern and Vaccination Efficiency in Uzbekistan." *J Med Virol* 80, no. 2 (2008): 217-24.

204. Gutiérrez, C., Devesa, M., Loureiro, C. L., León, G., Liprandi, F., and Pujol, F. H. "Molecular and Serological Evaluation of Surface Antigen Negative Hepatitis B Virus Infection in Blood Donors from Venezuela." *Journal of medical virology* 73, no. 2 (2004): 200-07.

205. Cardona, N. E., Loureiro, C. L., Garzaro, D. J., Duarte, M. C., Garcia, D. M., Pacheco, M. C., Chemin, I., and Pujol, F. H. "Unusual Presentation of Hepatitis B Serological Markers in an Amerindian Community of Venezuela with a Majority of Occult Cases." *Virol J* 8 (2011): 527.

206. Huy, T. T., Ushijima, H., Quang, V. X., Ngoc, T. T., Hayashi, S., Sata, T., and Abe, K. "Characteristics of Core Promoter and Precore Stop Codon Mutants of Hepatitis B Virus in Vietnam." *J Med Virol* 74, no. 2 (2004): 228-36.

207. Thuy Le, T. T., Ryo, H., Van Phung, L., Furitsu, K., and Nomura, T. "Distribution of Genotype/Subtype and Mutational Spectra of the Surface Gene of Hepatitis B Virus Circulating in Hanoi, Vietnam." *J Med Virol* 76, no. 2 (2005): 161-9.

208. Thuy, P. T. B., Alestig, E., Liem, N. T., Hannoun, C., and Lindh, M. "Genotype X/C Recombinant (Putative Genotype I) of Hepatitis B Virus Is Rare in Hanoi, Vietnam—Genotypes B4 and C1 Predominate." *Journal of medical virology* 82, no. 8 (2010): 1327-33.

209. Bui, T. T. T., Tran, T. T., Nghiem, M. N., Rahman, P., Tran, T. T. T., Dinh, M. N. H., Le, M. H., Nguyen, V. V. C., Thwaites, G., and Rahman, M. "Molecular Characterization of Hepatitis B Virus in Vietnam." *BMC Infect Dis* 17, no. 1 (2017): 601.

210. Trang, N. H., That, B. T. T., Thanh, T. T. T., Chau, L. N., Thanh, T. T., Ngoc, N. M., Hung, N. M., Chau, N. V. V., and Rahman, M. "A17 Molecular Characteristics of Hepatitis B Virus (Hbv) Isolated from Chronic Hepatitis B Patients in South Vietnam." *Virus Evol* 3, no. Suppl 1 (2017).

211. Sallam, T. A., and William Tong, C. "African Links and Hepatitis B Virus Genotypes in the Republic of Yemen." *Journal of medical virology* 73, no. 1 (2004): 23-28.

212. Chisenga, C. C., Musukuma, K., Chilengi, R., Zurcher, S., Munamunungu, V., Siyunda, A., Ojok, D., Bauer, S., Wandeler, G., Vinikoor, M., and For International Epidemiological Databases to Evaluate, A. I. S. a. I. "Field Performance of the Determine Hbsag Point-of-Care Test for Diagnosis of Hepatitis B Virus Co-Infection among Hiv Patients in Zambia." *J Clin Virol* 98 (2018): 5-7.

213. Vinikoor, M. J., Zürcher, S., Musukuma, K., Kachuwaire, O., Rauch, A., Chi, B. H., Gorgievski, M., Zwahlen, M., and Wandeler, G. "Hepatitis B Viral Load in Dried Blood Spots: A Validation Study in Zambia." *Journal of Clinical Virology* 72 (2015): 20-24.
