# Supplementary material for: The Global Hepatitis B Virus Genotype Distribution Approximated from Available Genotyping Data
Source: Genes (Basel). 2018 Oct 15;9(10):495. doi: 10.3390/genes9100495 (PMC6210291; doi:10.3390/genes9100495)
Supplement: Supplementary file 1 [file genes-09-00495-s001.zip › Supplementary files_new/Table S3 .docx]

**Table S3.** Geographical dispersal of HBV infections and genotypes

| **Continent & Region** | |  | **A** | **B** | **C** | **D** | **E** | **F** | **G** | **H** | **I** | **Total**  **HBV** |
| --- | --- | --- | --- | --- | --- | --- | --- | --- | --- | --- | --- | --- |
|  | |  | (% of global infections with respective genotype) | | | | | | | | |  |
| AFRICA | |  | 73.6 | 0.05 | 0.10 | 22.0 | 99.4 | 0.00 | 0.00 | 0.00 | 0.00 | 35.3 |
|  | *Eastern Africa* |  | 44.6 | 0.05 | 0.10 | 10.6 | 6.79 | 0.00 | 0.00 | 0.00 | 0.00 | 11.3 |
|  | *Middle Africa* |  | 12.8 | 0.00 | 0.00 | 0.19 | 13.7 | 0.00 | 0.00 | 0.00 | 0.00 | 4.62 |
|  | *Northern Africa* |  | 1.35 | 0.00 | 0.00 | 10.1 | 2.41 | 0.00 | 0.00 | 0.00 | 0.00 | 3.02 |
|  | *Southern Africa* |  | 7.80 | 0.00 | 0.00 | 0.41 | 0.45 | 0.00 | 0.00 | 0.00 | 0.00 | 1.49 |
|  | *Western Africa* |  | 7.01 | 0.00 | 0.00 | 0.76 | 76.1 | 0.00 | 0.00 | 0.00 | 0.00 | 14.9 |
| ASIA | |  | 17.2 | 98.8 | 98.6 | 61.9 | 0.19 | 0.23 | 0.00 | 0.00 | 100 | 57.9 |
|  | *Central Asia* |  | 0.88 | 0.00 | 0.05 | 6.91 | 0.00 | 0.00 | 0.00 | 0.00 | 0.00 | 1.69 |
|  | *Eastern Asia* |  | 0.41 | 61.0 | 76.6 | 5.00 | 0.00 | 0.13 | 0.00 | 0.00 | 94.0 | 30.5 |
|  | *South-Eastern Asia* | | *4.89* | 5.36 | 36.1 | 17.0 | 0.13 | 0.00 | 0.00 | 0.00 | 0.00 | 6.05 |
|  | *Southern Asia* |  | 9.46 | 1.73 | 4.99 | 37.2 | 0.05 | 0.00 | 0.00 | 0.00 | 0.00 | 12.1 |
|  | *Western Asia* |  | 1.13 | 0.00 | 0.03 | 12.7 | 0.14 | 0.10 | 0.00 | 0.00 | 0.00 | 3.10 |
| EUROPE | |  | 3.90 | 0.17 | 0.27 | 13.5 | 0.14 | 1.88 | 9.44 | 3.25 | 0.00 | 4.00 |
|  | *Eastern Europe* |  | 1.94 | 0.01 | 0.09 | 9.76 | 0.00 | 0.12 | 0.00 | 1.93 | 0.00 | 2.70 |
|  | *Northern Europe* |  | 0.09 | 0.04 | 0.02 | 0.14 | 0.01 | 0.01 | 0.55 | 0.00 | 0.00 | 0.06 |
|  | *Southern Europe* |  | 1.08 | 0.03 | 0.07 | 2.74 | 0.06 | 1.43 | 4.23 | 1.33 | 0.00 | 0.85 |
|  | *Western Europe* |  | 0.79 | 0.09 | 0.09 | 0.86 | 0.07 | 0.32 | 4.66 | 0.00 | 0.00 | 0.38 |
| LATIN AMERICA/CARIBBEAN | | | 4.51 | 0.05 | 0.11 | 1.96 | 0.19 | 97.7 | 73.5 | 95.2 | 0.00 | 2.20 |
|  | *Caribbean* |  | 2.59 | 0.00 | 0.00 | 0.65 | 0.19 | 0.00 | 0.00 | 0.70 | 0.00 | 0.61 |
|  | *Central America* |  | 0.09 | 0.00 | 0.01 | 0.03 | 0.00 | 4.33 | 23.8 | 94.5 | 0.00 | 0.13 |
|  | *South America* |  | 1.83 | 0.04 | 0.10 | 1.29 | 0.00 | 93.4 | 49.7 | 0.00 | 0.00 | 1.45 |
| NORTHERN AMERICA | | | 0.77 | 0.83 | 0.47 | 0.25 | 0.04 | 0.16 | 17.1 | 1.58 | 0.00 | 0.44 |
| OCEANIA * | |  | 0.02 | 0.14 | 0.42 | 0.30 | 0.00 | 0.00 | 0.00 | 0.00 | 0.00 | 0.20 |

Numbers are rounded to 3 significant digits and represent % of global infections with respective HBV genotype in the indicated world region. * Oceania includes pooled data of Australia/New Zealand, Melanesia, Micronesia, Polynesia
