# Supplementary figures and images for: The Global Hepatitis B Virus Genotype Distribution Approximated from Available Genotyping Data
Source: Genes (Basel). 2018 Oct 15;9(10):495. doi: 10.3390/genes9100495 (PMC6210291; doi:10.3390/genes9100495)

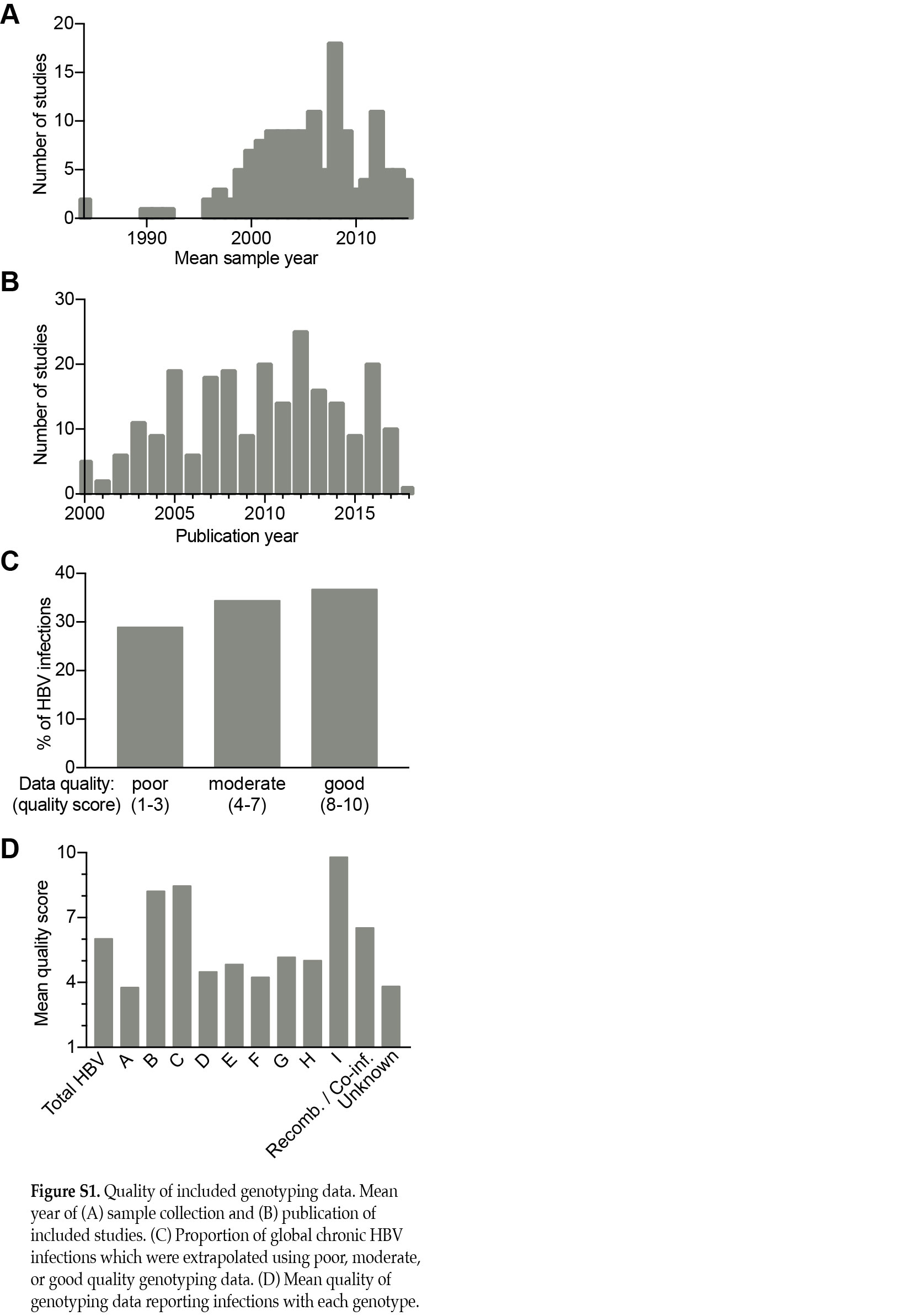

Supplement: Supplementary file 1 [file genes-09-00495-s001.zip › Supplementary files_new/Figure S1.jpg]
